# Growth and Developmental Processes Alternate During Larval Development of Atlantic Herring

**DOI:** 10.1111/ede.70022

**Published:** 2025-11-22

**Authors:** Vivian Fischbach, George P. Franz, Timo Moritz, Daniela Ohde, Philipp Thieme, Paul Kotterba, Patrick Polte, Bianka Grunow

**Affiliations:** ^1^ Aquatic Ecology, Institute of Biosciences University of Rostock Rostock Germany; ^2^ Ocean Museum Germany Stralsund Germany; ^3^ Thünen Institute for Baltic Sea Fisheries Rostock Germany; ^4^ Work Group “Fish Growth Physiology”, Research Institute for Farm Animal Biology (FBN) Dummerstorf Germany; ^5^ Zentrum für Taxonomie und Morphologie, Leibniz‐Institut zur Analyse des Biodiversitätswandels Hamburg Germany; ^6^ Work Group “Endocrinology of Farm Animals”, Research Institute for Farm Animal Biology (FBN) Dummerstorf Germany

**Keywords:** *Clupea harengus*, larval growth, myogenesis, ontogeny, skeletal development

## Abstract

During their development, fish pass through a series of developmental processes advancing, for example, their physiological and locomotive abilities. In particular, many marine fish larvae often hatch at an (semi‐) embryonic developmental stage, and existential processes, such as digestion and respiration, and structures, such as muscle and skeleton, must form and/or change during the larval development. In this study, we analyzed the gene expression of factors associated with myogenesis, skeletogenesis, and growth within the different larval life stages of Atlantic herring. We evaluated these results in relation to length and stage ratio and performed histological analysis of cross‐sections of herring larvae in different stages. Overall, the length per stage ratios showed that there are two major growth periods in larval herring development, the first occurring during the dorsal fin development phase and the second during the transition from caudal fin to pelvic fin development phase. This is consistent with the histological analysis, as an increase in muscle fibers was observed in both phases. The gene expression data also showed that factors responsible for muscle cell lineage determination and fiber development were highest before a period with increased growth. Combining our results with other studies on skeletogenesis, organogenesis, and the development of neural and sensory systems in herring, it becomes evident that other energetically costly developmental processes tend to occur in periods when growth is less prominent. It can therefore be concluded that growth and developmental priority periods alternate during larval development.

## Graphical abstract

During their development, fish pass through a series of developmental processes advancing, for example, their physiological and locomotive abilities. Gene expression analysis combined with histological and morphological analysis of Atlantic herring in different larval life stages showed two major growth periods in larval herring development alternating with periods of increased development, for example, of the skeleton.

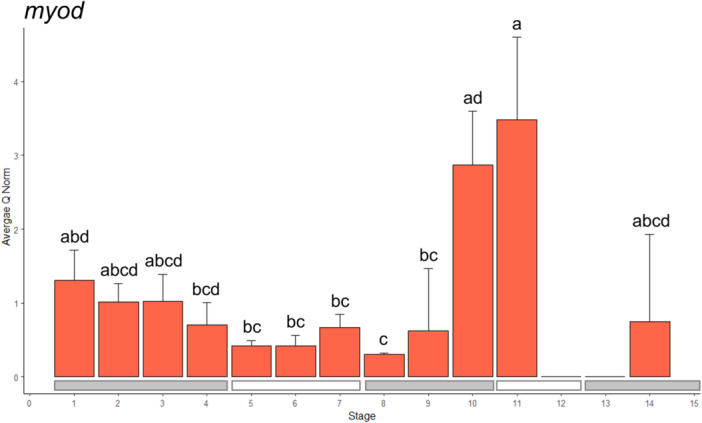

## 1 Introduction

Many marine fish larvae hatch at an early stage of development, and many physiological and behavioral processes are not yet fully developed or differ from those observed in adults (igf1 x1: Miller and Kendall 2009). The development of swimming abilities is of crucial importance during fish ontogeny, as it is directly related to distinct survival advantages for larvae. These include an increased foraging capacity (Munk and Kiøboe 1985), an enhanced ability to avoid predators (Yin and Blaxter 1987), and impacts on larval dispersal and migration (Miller and Kendall 2009). Most marine fish larvae are unable to swim immediately after hatching and initially behave more like plankton particles (Voesenek et al. 2018). By further development of muscle tissue and skeletal elements, as well as the depletion of the yolk sac, the larvae eventually gain swimming capability.

In fish, the development of muscle tissue occurs already during embryonic development with the onset of somitogenesis. Later, the somites start to differentiate into myogenic precursor cells (the myoblasts) (Seale et al. 2000). Determination of the muscle cell lineages is initiated and controlled by multiple myogenic regulatory factors (Berkes and Tapscott 2005; Francetic and Li 2011), such as myogenic factor 5 (*myf5*) and myogenic differentiation factor (*myod*), which are commonly regulated by paired box genes 3 and 7 (*pax3, pax7*) (Bendall et al. 1999; Buckingham 2007; Buckingham and Relaix 2007; Koganti et al. 2020). Myoblasts then differentiate into myotubes and finally into mature muscle tissue (Watabe 1999). During this process, myogenin and myogenic factor 4 (*mrf4*, *MYF6* in humans) are responsible for the terminal differentiation and fusion of myoblasts into myotubes (Tajbakhsh et al. 1997). Myogenesis also involves an extensive control network with genes such as myocyte‐specific enhancer factor 2A (*mef2a*), which can upregulate myogenin transcription (Johnston and Hall 2004).

The development of muscle tissue enables fish to grow, either by increasing the number of cells (hyperplasia) or by increasing the size of the cells (hypertrophia) (Churova et al. 2017). It is generally accepted that larger fish larvae have better chances of survival because they can swim faster, and thus escape predators and catch prey more easily (“Bigger is better”) (Bailey and Batty 1983; Blaxter and Batty 1985; Hubbs and Blaxter 1986; Eaton and Didomenico 1986). Therefore, growth and the increase in size are determining factors during larval development. Growth in fish is strongly regulated by environmental factors such as temperature, food availability, and photoperiod (Miller and Kendall 2009). On the gene level, growth and development are mainly regulated by growth hormone (*gh*). It promotes growth by stimulating the liver to release insulin‐like growth factors (*igfs*) like insulin‐like growth factor 1 (*igf1 x1*). *igf1 x1* and insulin regulate myogenesis by binding to specific receptors and initiating cascades that enhance myogenic proliferation and differentiation, as well as control muscle regeneration (Koganti et al. 2020). In teleost fish, *gh* is the major activator for the *igf1 x1* system and stimulates *igf x1* expression in various tissues (Moriyama and Kawauchi 2001). Growth hormone also has important metabolic effects, and in fish, a strong link between energy status and circulating plasma GH levels exists (Moriyama and Kawauchi 2001).

In addition to myogenesis and growth, skeletal development plays an important role in the ontogeny of fish larvae, as new features such as the caudal fin develop, which can increase swimming ability and/or swimming speed. Two main processes take place during skeletogenesis. First, histogenesis (the histological differentiation of skeletal tissues) involves the specialization and proliferation of different cell types, such as chondrocytes and osteoblasts, which arise from mesenchymal condensations of skeletogenic cells. This process is often induced by factors such as the bone morphogenetic proteins (BMPs) (Urist et al. 1979; Wozney et al. 1988). Secondly, morphogenesis (the acquisition of skeletal elements, their location, shape, and size) takes place (Eames et al. 2012).

The Atlantic herring is common in temperate marine ecosystems and is considered a keystone species as it occupies an important role in pelagic food webs, for example, as major prey for many other commercial fish species (Checkley et al. 2009). Furthermore, it is the fourth most exploited fish species in the world (FAO 2024). To monitor the adult stock population, herring larval surveys have been established to survey the abundance of larvae in the North Sea and Baltic Sea. In most cases, such as for the western Baltic spring spawning herring, larval abundance has declined in recent decades (ICES 2023), and several factors have been identified that negatively affect herring development (Moyano et al. 2023). The majority of studies concentrate exclusively on the impact of environmental factors, without considering the transformations and associated functional changes of the larvae. However, the functional morphology may exert a significant influence on larval survival, and thus requires detailed investigation.

Herring has a very distinct larval ontogeny, and newly hatched larvae do not possess a cartilaginous or bony skeleton within the post‐cranial skeleton (Fischbach et al. 2022, 2023). Their larval development can be grouped into four major larval phases (yolk sac, dorsal fin development, caudal fin development, and pelvic fin development), which are all subdivided into 12 larval stages leading to the juvenile phase. These 12 stages were defined by specific developmental milestones: Stages 1–4 focus on the degree of yolk sac consumption, Stages 5–7 involve the development of the dorsal fin, Stages 8–10 track the notochord flexion and development of the anal fin, and Stages 11–12 cover the development of the pelvic fin and overall changes in body morphology before metamorphosis. The juvenile phase can then be grouped into three stages (13–15) in which squamation and ossification of some postcranial elements occur (Fischbach et al. 2023). Some of these developmental stages can constitute developmental bottlenecks. For example, the first feeding stage (Stage 4) is susceptible to high mortality rates if no suitable prey organisms are available (Stenevik et al. 2021). A deeper understanding of the interactions between different developmental processes during larval development, in conjunction with environmental parameters, enables the identification of mortality bottlenecks and the determination of limiting environmental factors during these developmental periods.

In herring, the muscle development also starts in the embryonic development with somitogenesis occurring after completion of epiboly (Hill and Johnston 1997). Three distinct phases of muscle formation can be distinguished (Johnston et al. 1998). First, the process of formation of embryonic muscle fibers. Under the electron microscope, myotomes of freshly hatched herring larvae typically contain a single superficial layer of embryonic slow muscle fibers surrounding a larger number of embryonic fast muscle fibers (Johnston and Hall 2004). The initial increase in the muscle's diameter thus largely involves the hypertrophy of embryonic muscle fibers, which were formed before hatching. The second phase involves distinct germinal zones of myoblasts at the dorsal and ventral ends of the myotome. Johnston et al. (1998) found in herring from the Scottish Clyde Sea stock that these proliferative zones became exhausted at 22–25 mm TL. Lastly, the third phase of myogenesis involves the general activation of myoblasts scattered throughout the myotome. In herring, the number of muscle fibers begins to increase at larger body lengths, that is, around 22–28 mm TL (Johnston et al. 1998). During the juvenile phase, most, if not all, myogenic precursor cells are incorporated into the basal lamina of muscle fibers and form the satellite cell population, as described for European seabass (Veggetti et al. 1990). In fish larvae, hyperplasia and hypertrophy are responsible for the resulting growth (Churova et al. 2017). After the initial formation of muscle cells, hyperplasia also occurs in the adult stages, and thus fish exhibit a lifelong muscle growth in contrast to terrestrial vertebrates (Watabe 1999).

### 1.1 Aim of This Study

The present study examines the expression of genes related to various developmental processes during the larval and juvenile stages of herring, in conjunction with data on growth and histology of muscle development. The objective is to gain a deeper comprehension of the larval ontogeny and the capabilities of the larvae at different developmental stages. It is hypothesized that throughout the course of development, distinct biological processes will be prioritized in accordance with both environmental pressures and the developmental requirements faced by the larvae at each respective stage. By combining different methodologies and data on developmental processes, we aim to characterize the different larval stages of herring.

## 2 Materials and Methods

### 2.1 Sampling Procedure

#### 2.1.1 Sampling Area

Greifswald Bay (54°13′22″ N, 13°32′48″ E), as the largest shallow coastal water body of the Western Baltic Sea, constitutes a major spawning ground for the western Baltic herring (Moll et al. 2019). It has an average depth of 5.8 m, a total area of 514 km^2^, and a water volume of 3 billion m^3^ (Stigge 1989). It is located on the coastline of the state of Mecklenburg‐Western Pomerania and is limited to the north by the Island of Rügen. The Baltic Sea is connected to Greifswald Bay via the narrow Strelasund in the west and via the Pommeranian Bight in the east (Seifert 1938). Nevertheless, only a limited amount of water exchange occurs with the open Baltic Sea, primarily due to the influence of strong winds (Hammer 2009).

#### 2.1.2 Sampling Methods

Herring larvae of the western Baltic spring spawned stock were sampled fortnightly as part of the Rügen Herring Larvae Survey (RHLS) of the Thünen Institute of Baltic Sea Fisheries in the spawning season of 2023. Larvae were caught with a bongo net (diameter 60 cm, mesh size 335 µm) towed at two knots in a double oblique haul at 36 stations in the Greifswald Bay area and the Strelasund. Additionally, samples of juvenile life stages of Western Baltic Spring spawning herring (Stages 14) were caught using a beach seine (6.9 m opening, 1.2 m height, 2 mm mesh size) along the shoreline of Greifswald Bay and the adjacent outer coastline in May to June. Collection permits were obtained from the Landesamt für Landwirtschaft, Lebensmittelsicherheit und Fischerei Mecklenburg‐Vorpommern (LALLF, 03.12.2022, file number 7308.5). The larvae were unable to survive the catching process due to their inherent fragility. This also applied to most juvenile herring caught via beach seine. Juvenile herrings that survived the catch were euthanized with an overdose of clove oil. This study followed international, national, and institutional guidelines for animal treatment and sacrifice and complied with Directive 2010/63/EU and the German Animal Welfare Act [§ 4(3) TierSchG].

For length measurements, larvae were fixed in 4% buffered formaldehyde. To determine the length range per developmental stage, a maximum of 30 larvae per developmental stage were measured to the nearest 0.5 mm from station 207 of the survey for cruises 1–17 (Table S1). The known shrinkage of larvae in 4% buffered formaldehyde was not corrected.

For the analysis of gene expression, the larvae were sorted into developmental stages following Fischbach et al. (2023) right after catching. Larvae within the same developmental stages were pooled (max. *n* = 30) and fixed in RNA*Later*. The sampling was stopped after the necessary number of larvae per day was sampled. Consequently, larvae for this study were sampled at different stations throughout the area, with the objective of ensuring rapid fixation, given the time required for processing the larvae (on average, fixation occurred 30 min after the start of the haul). The samples were then stored at −80°C for later use.

To conduct supplementary histological analysis, herring larvae obtained during the RHLS in 2021 and stored in 4% buffered formaldehyde were utilized. One larva of different developmental stages was selected, and histologically processed to examine muscle tissue. Additionally, samples of juvenile herring (Stage 14) obtained via beach seine in 2023 and preserved in 2% paraformaldehyde were included in the study.

#### 2.1.3 Selection of Samples for the Genetic Analysis

For this genetic analysis, larvae within the different developmental stages that occurred earliest in the season and in sufficient quantities were used. To balance the scientific requirements for RNA isolation with ethical considerations according to the 3 R principle, as few individuals as possible were used while ensuring sufficient biological material. For each developmental stage, three replicate pools were sampled, each consisting of 3–30 larvae. To accommodate for a necessary amount of sampling material for RNA‐isolation, as well as the varying abundance of the different developmental stages, the number of larvae per pool decreased for the later developmental stages (Table 1). In earlier stages, larvae were abundant, and up to 30 individuals per pool needed to be used to obtain the necessary mRNA quantity for gene expression analysis. In contrast, in later stages, larval size increased while the abundance decreased, and accordingly, the number of larvae per pool was reduced. For each developmental stage, the sample size per pool was identical. Stages 12, 13, and 15 had to be excluded from the analysis as the number of sampled larvae was insufficient during the 2023 season.

**Table 1:** Overview of the number of replicate pools, sample size per replicate pool, total number of larvae sampled, catch method, and cruise where larvae were sampled in a specific developmental stage.

Stage	Replicate pools	*n* per replicate pool	Total *n* of larvae sampled	Catching device	Cruise
1	3	30	90	Bongo net	5
2	3	30	90	Bongo net	5
3	3	30	90	Bongo net	5
4	3	30	90	Bongo net	5
5	3	20	60	Bongo net	9
6	3	20	60	Bongo net	9
7	3	6	18	Bongo net	9
8	3	6	18	Bongo net	12
9	3	3	9	Bongo net	12
10	3	3	9	Bongo net	15
11	3	3	9	Bongo net	15
14	3	3	9	Beach seine	—

### 2.2 Analysis of Gene Expression

#### 2.2.1 Selection of Genes and Primer Design

For the analysis, 20 genes were selected (Table 2) with a focus on regulation of myogenesis (*mef2a*, *myod*, *myog*; *mrf4, myh6*), skeletal and structural development (*bmp4, col1a, mustn1, sparc*), general development (*fgf6*, *pax3a*, *pax7*, *paxbp1*), and growth (*gh, ghrb, igf1 x1, igfra*). Following the MIQE guidelines (Bustin et al. 2009), three reference genes (*eef1a1, rpl32, rps5*) were chosen to allow the normalization of the expression data (Pfaffl 2001; Swirplies et al. 2019), and corresponding primers were either designed as described below or taken from the literature, for example, *eef1a1* (Olsvik et al. 2011) and *igf1 x1* (Joly et al. 2023).

**Table 2:** List of genes and corresponding primers analyzed in this study.

	Gene		Forward primer	Reverse primer	Genbank reference	bp
1	*igf1 x1*	Insulin growth factor 1 X1	GACAGCACATGGTACACTTGA	CCTGCGCAATGGAACAAAGT	XM_012830244.3	132
2	*igf1ra*	Insulin growth factor 1 receptor a	CAAGCCCTGGACGCAGTACG	TTGGCATAGGCCCTGGCATCA	XM_031564413.2	83
3	*gh*	Growth hormone	CTCTCACTGCATCTCGGACTC	TGAAGGCCTGGCTCGGATACT	XM_031567293.2	49
4	*ghrb*	Growth hormone receptor b	TCCTGACATGGCAGCTATCCAA	TGATACACAGCCAGTCAAGTAAG	XM_012815656.3	58
5	*fgf6*	Fibroblast growth factor 6	ATCCTCCCAGACGGCAGTATAA	TTGTTTGGCAGCAAGGTCTCGT	XM_012823561.3	117
6	*pax3a*	Paired box 3a	GTCCAAAATCCTGTGCAGGTAC	CTTGTCTATGTCGGGCGTTGTA	XM_031560078.2	63
7	*pax7*	Paired box 7	AGTTTCCCACGGTTGTGTATCC	GCGCTTGTACTCCTCGATTCTT	XM_012820838.3	49
8	*paxbp1*	Paired box binding protein 1	GAGAAAAAGCGGGCCAAGGATA	AGAAGCCGCATCAGTGCTTGTA	XM_012827707.3	120
9	*sparc*	Secreted protein acidic and rich in cysteine	ACTCCAGCTGACTTCTGACCG	GCCAGGCACAGGAGAAAGACA	XM_012834436.3	52
10	*bmp4*	Bone morphogenetic protein 4	CCGGAGGCCAACAAACATGGA	GCAGGACGTGGCATAGCAAAAT	XM_053335823.1	26
11	*mustn1*	Musculoskeletal, embryonic nuclear Protein	AAAGGGGCCCGCAGCAAGCT	ACACCGAAGGAGCCACCTTAC	XM_012841214.3	66
12	*col1a*	Pro‐alpha1 chains collagen	ACATACAGCGTGGTCGAGGATA	TCCATAGGAGCGATGTCAATAATA	XM_042705789.1	55
13	*mrf4*	Myogenic factor 6	ATTCCAATGCGCCAATGACGAG	CTGTTCATTTTCCCGAGACATCT	XM_012841355.3	63
14	*myod*	Myoblast determination protein 1	AGACAACTATTACCCAGTGATCG	AGACAGTCCAGACTGGAAATAAC	AF265553.1	130
15	*myog*	Myogenin	ACCACTGTGCCACCGCGTAC	AGTGGTGTTGGCGTCCGTGAT	XM_012832005.3	48
16	*mef2a*	Myocyte‐specific enhancer factor 2 A	CGGCATGGCAACAGCACCAAT	GAGAGATGGGCTCTGACTTGAT	XM_031564423.2	56
17	*myh6*	Myosin heavy chain 6	GACCTGGAACGTGGCAAAAGAA	CCATCTTCAACTTTGCTGCTTAG	XM_042709945.1	75
18	*eef1a1*	Eukaryotic translation elongation factor 1 alpha 1	TCTGTGGAGATGCACCATGAG	ACGTTGCCACGACGGATATC	DQ334852.1	57
19	*rpl32*	Ribosomal protein L32	AAAGAAGTTCATCAGGCATCAATC	CTTGTTGCTACCATAACCAATGTT	XM_012822682.3	55
20	*rps5*	Ribosomal protein S5	TTGTCAAGCACGCCTTCGAGAT	GACCGATACGAGTGGAGTCCT	XM_012831474.3	40

Sequences for the primer design were obtained from the NCBI GenBank database. If available, gene sequences from herring were used. Otherwise, sequences from other clupeomorph (mostly denticle herring, *Denticeps clupeoides* Clausen, 1959) or otomorph (mostly Zebrafish: *Danio rerio* (Hamilton, 1822)) species were used. The oligonucleotide primer sequences (Table 2) were designed using the software PSQ‐Assay Design (Version 1.0.6, Biotage) and synthesized by SIGMA–Aldrich (Merck). Designed primers were evaluated for their effectiveness through PCR gel electrophoresis and qPCR trials. This testing ensured the primers were suitable for use by preventing the formation of primer dimers or multiple product bands. For all genes, the target region amplified in the analysis was sequenced (Genewiz, Azenta Life Sciences). The forward and reverse sequences were aligned in MEGA (Version 7.0.26) and blasted in GenBank to confirm that the intended gene was indeed targeted by the primers.

#### 2.2.2 RNA Extraction and Fluidigm PCR

As described above, larvae were combined into three different pools within the same developmental stages. Each pool of larvae was mechanically homogenized (Precellys Evolution, Bertin technologies) and RNA was isolated with a Trizol‐Chloroform precipitation. The total extracted RNA was purified using the RNeasy Micro Kit (Qiagen) according to the manufacturer's instructions. RNA concentration and quality were quantified using the NanoDrop One (Thermo Scientific) spectrophotometer using the ratio of absorbance between 260 and 280 nm. Until further use, the RNA was stored at −80°C. RNA was reverse‐transcribed into cDNA by using the iScript cDNA Synthesis Kit.

For a high‐throughput analysis of the gene expression of the different sample groups, a transcriptional analysis was conducted using a quantitative real‐time PCR based on a microfluid circuit system (Biomark/Standard BioTools) using EvaGreen fluorescence dyes (Bio‐Rad) after the protocol of Rebl et al. (2019). The design of Fluidigm 48.48 Dynamic Array IFC allows the parallel quantification of 48 transcripts in 48 samples each. Three biological replicates were assessed, and two technical replicates of each gene section were made. The raw cycle thresholds (CT‐values) were retrieved using the real‐time PCR analysis software (Fluidigm, Version 3.0.2), and the mean cycle thresholds for both technical replicates were calculated and used for further analysis. For all gene expression data, all three pools were available for analysis, except for Stage 14 in *pax3a*, *paxbp1*, and *myod*, where only the expression data of two pools were available. Also, unfortunately, no expression data of *igf1ra* for Stage 14 were available. Contrary to the other genes, the expression of *gh* was analyzed by using CT values obtained from LightCylcer PCR (program: 1× cycle: 4 min at 94°C, 40× cycles: 30 s at 94°C, 30 s at 60°C, 30 s at 72°C, 1× cycle: 7 min at 72°C). For *gh*, two identical PCRs were conducted, and one PCR was conducted for each housekeeping gene. The analysis of the CT‐values followed the above‐mentioned protocol; however, no adjustment between the different runs occurred.

#### 2.2.3 Statistical Data Analysis and Graph Design

For the length comparisons, size ranges were compared by a Wilcox Test followed by a Dunn's Test (Tables S3 and S4). Additionally, an Analysis of Variance (ANOVA) was performed following the guidelines by Underwood (1996). For the expression data, the CT‐values were processed according to Pfaffl (2001). To compare expression levels between the developmental stages, an ANOVA was conducted via R (version 4.4.2, package ucrt) followed by a Tukey post hoc test. The significance level for the statistical test was defined at *p* = 0.05. Non‐normal distributed data were transformed by logarithm or square root, depending on the degree of skewness. Following the recommendations by Underwood (1996), an ANOVA was conducted even in cases where the data were neither normally distributed nor showed homogeneity of variances. To investigate correlations between the gene expression levels, a robust Pearson correlation analysis was conducted between the mean expression rate per stage and genes (WRS2 package: Mair and Wilcox 2020). All graphs and figures were generated using the ggplot2 library in R.

### 2.3 Histology

#### 2.3.1 Histological Sample Preparation

Body cross sections were cut close to the anus for each larval stage. Due to the delicacy of herring larvae, especially in the early developmental stages, larvae up to Stage 6 were initially embedded in 2% agarose (Biodeal, #CH1001,0500, Charge T934937) before cross‐sections were taken. All larvae were decalcified using an EDTA and NaOH solution adjusted to a pH of 7.2, adapted from the protocol of Warshawsky and Moore (1967). The duration of decalcification varied with developmental stage and took two (Stages 1–6) to 7 days (Stages 7–11, 14). Afterwards, the larvae were dehydrated within a stepwise alcohol gradient in the Intelsint TP300 Tissue Processor. Again, the program was adjusted to the developmental stage (Table S2). Larvae of Stages 7 and up were only then cut close to the anus, and all larvae were embedded in paraffin (paraffin station MPS/P2, SLEE Mainz, Germany). Tissue was cut into 5 µm thick cross‐sections via microtome (pfm Rotary 3006 Em, pfm medical) and transferred to glass slides. Best results of deparaffinization were achieved with an incubation at 65°C for 1 h and then subsequently submerging the slices in Roticlear (2*5 min), 99% Isopropanol (2*3 min), 70% Isopropanol (1*3 min), 50% Isopropanol (1*3 min), and distilled water (1*3 min). Finally, the sections were stained with hematoxylin and eosin and mounted with Roti‐Mount (ROTH 2848.2). Sections were examined and photographed using a Zeiss Axio Observer Z1 microscope (Carl Zeiss, Oberkochen, Germany) with 2.5× and 10× magnification. Final figures were compiled using Adobe Illustrator CC (version: 20.0.5).

## 3 Results

### 3.1 Length Ranges Per Stage (Figure 1)

**Figure 1:**
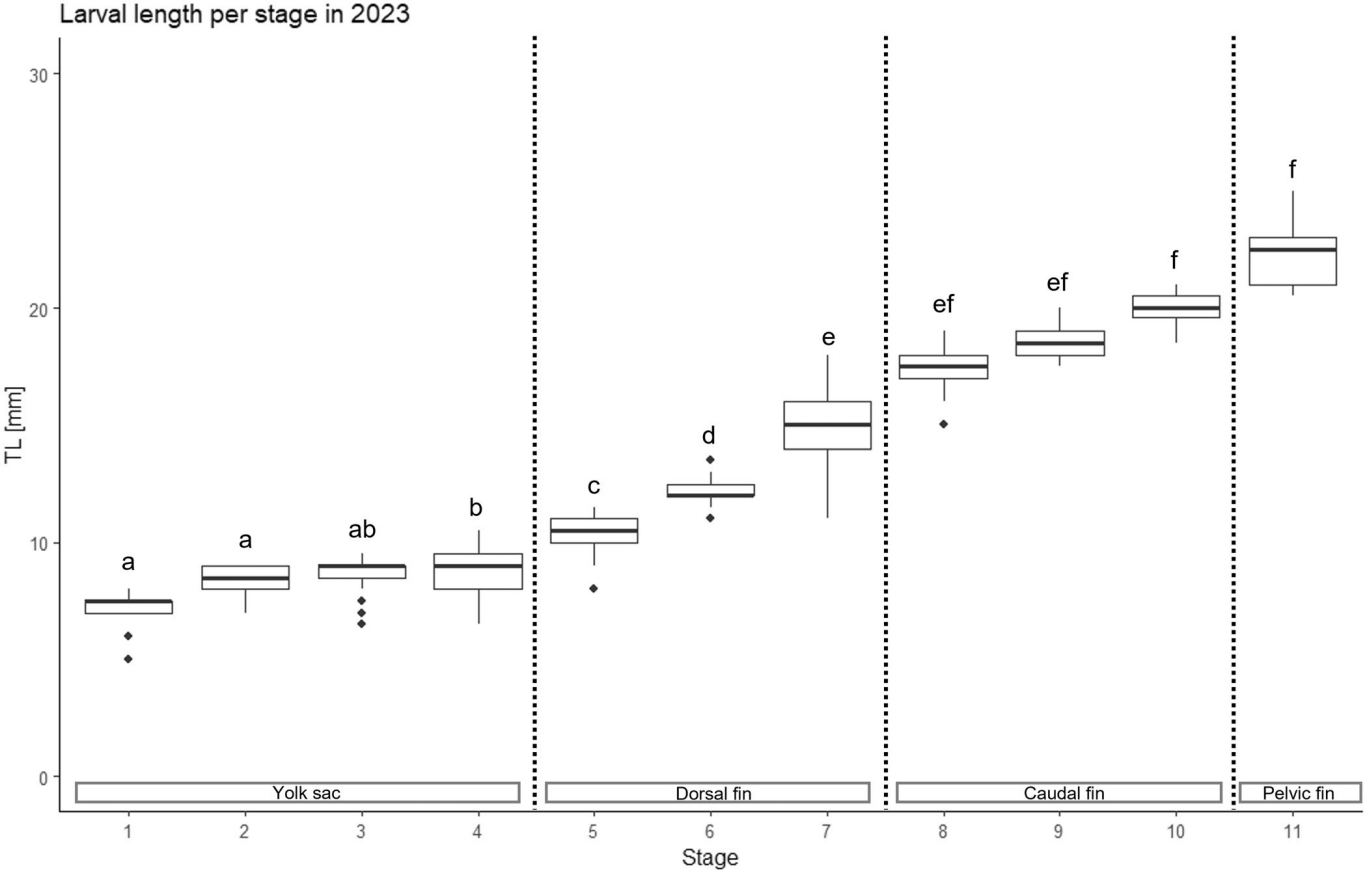
Length ranges per larval developmental divided in developmental phases (yolk sac, dorsal fin development, caudal fin development, and pelvic fin development phases), indicated in the rectangles at the bottom. The boxplots show the median, lower quantile (25%), and upper quantile (75%). The whiskers show the smallest and largest total lengths per developmental stage, and the dots represent outliers (= observations which is 1.5 times the interquartile range greater than the third quartile or 1.5 times the interquartile range less than the first quartile). The letters above the bars indicate which developmental stages do not show significant differences for *p* ≤ 0.05 (same letter) and which show significant differences (different letter).

Stages 7 and 11 showed the largest length per stage ranges, followed by Stage 4, suggesting that in these stages, increased growth occurred. Significant differences between the length ranges per stage were found (*p*‐value ≤ 2.2e^−16^, Tables S3 and S4). An analysis of the developmental phases revealed that during the yolk sac stages, the larvae were similar in size, and no significant differences were found between Stages 1–3. However, during the dorsal fin stages, there was a marked increase in growth rate, and the length per stage differed significantly between Stages 5–7. During the caudal fin development phase, although the larvae continue to grow, the rate of growth becomes less pronounced, and again, no significant differences were found in the length ranges between Stages 7–9 and between Stages 8–11.

### 3.2 Gene Expression

#### 3.2.1 Genes Involved in Regulation of Developmental Processes (Figure 2)

**Figure 2:**
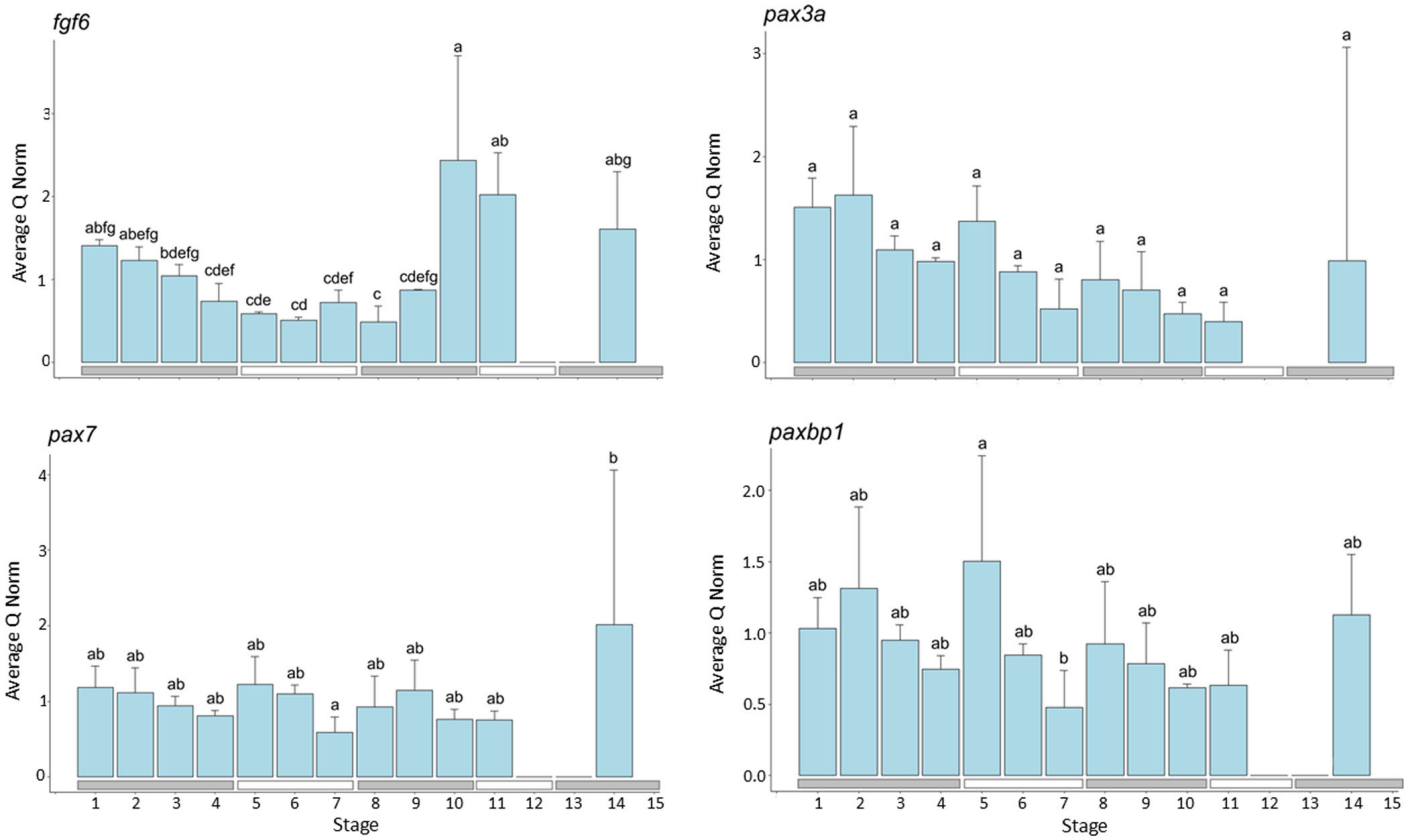
Expression of genes related to general developmental processes (*fgf6, pay3a, pax7, paxbp1*). The blue bars indicate the average normed quantity of detected cDNA in *n* = 3 pools of larvae within each development stage (exception: for Stage 14 of *pax3a* and *paxbp1*, only two pools were available), while the error bars indicate the positive standard deviation. The letters above the bars indicate which developmental stages do not show significant differences for *p* ≤ 0.05 (same letter) and which show significant differences (different letter). [Color figure can be viewed at wileyonlinelibrary.com]

The expression of *fgf6* shows significant differences between the developmental stages (*F*_24,11_ = 12.63, *p* = 1.85e^−07^, Tables S3 and S5). Initially, *fgf6* expression decreases, resulting in significant differences between Stages 1 and 5, as well as between Stages 6 and 8. After Stage 9, a steep increase in expression occurred, resulting in significant differences between Stages 10 and 11 compared to earlier stages (Stages 10 vs. 3–9, Stages 11 vs. 4–9).

The expression levels of *pax3a* differed among the developmental stages; however, in the post hoc test, no significant differences could be identified between the different stages (*F*_23,11_ = 2.571, *p* = 0.0271, Tables S3 and S6). Generally, the expression of *pax3a* generally decreases throughout the larval development of herring, reaching its lowest point at Stage 11. Therefore, the expression levels between Stages 1, 2, and 11 presumably account for most of the significance between the developmental stages.

The expression level of *pax7* differed significantly between larval and juvenile development (*F*_24,11_ = 2.324, *p* = 0.0408, Tables S3 and S7). While *pax7* expression levels were unaltered throughout larval development, it was highest during juvenile development.

The expression of *paxbp1* showed significant differences (*F*_23,11_ = 2.64, *p* = 0.0239, Tables S3 and S8) between Stage 5 compared to Stage 7.

#### 3.2.2 Genes Involved in Muscle Development (Figure 3)

**Figure 3:**
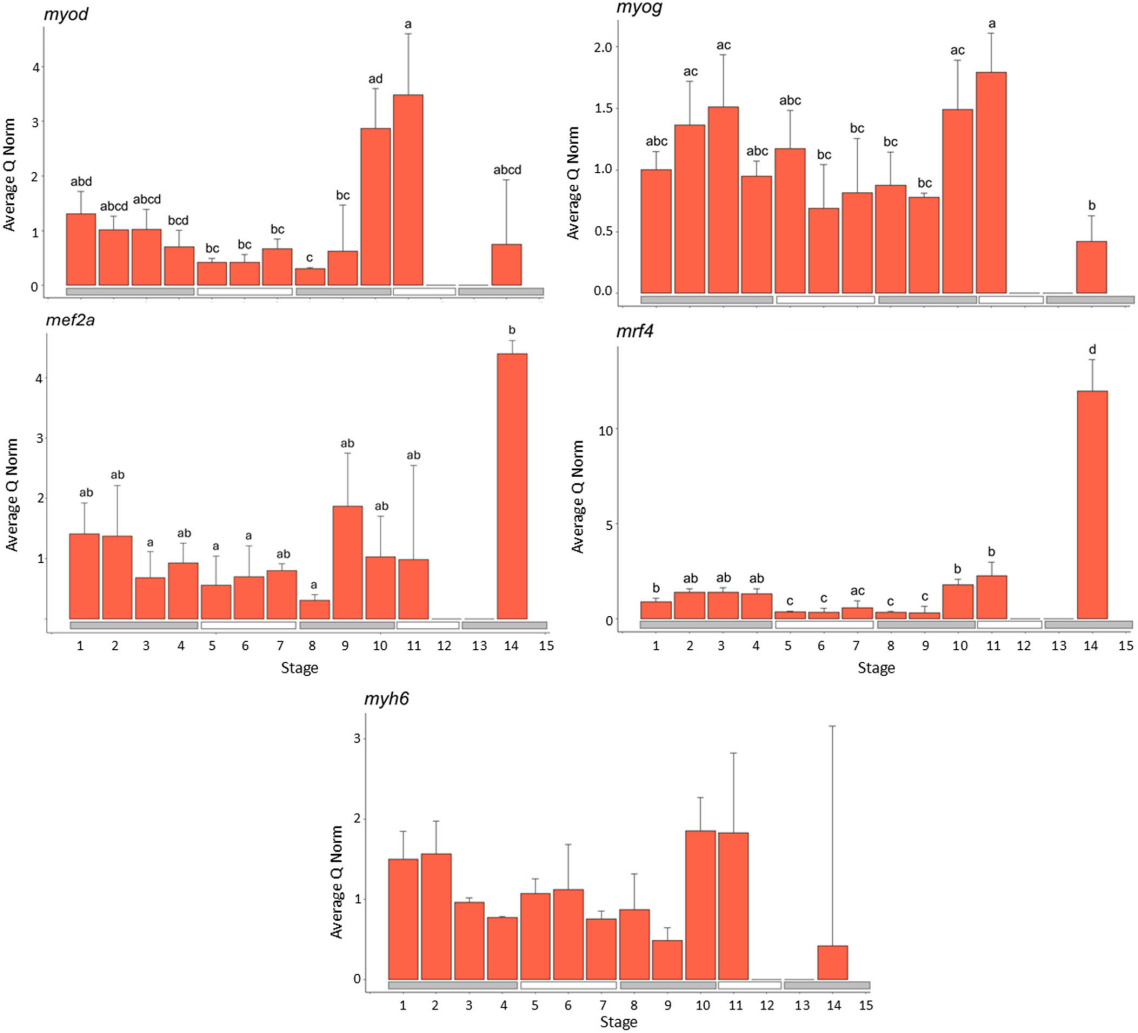
Expression of genes related to muscle development (*myod, myog, mef2a, mrf4, myh6*). The red bars indicate the average normed quantity of detected cDNA in *n* = 3 pools of larvae within each development stage (exception: for Stage 14 of *myod*, only two pools were available), while the error bars indicate the positive standard deviation. The letters above the bars indicate which developmental stages do not show significant differences for *p* ≤ 0.05 (same letter) and which show significant differences (different letter). If no difference was detected within the expression of the different stages, letters are not present. [Color figure can be viewed at wileyonlinelibrary.com]

The expression of *myod* showed significant differences between the developmental stages (*F*_23,11_ = 6.83, *p* = 5.58e^−05^, Tables S3 and S9). Expression during the earliest developmental stages (1–3) was moderate compared to the later stages. In Stages 10, 11, and 14, the expression levels significantly increased again and reached their peak at Stage 11.

The expression of *myog* also showed significant differences between the developmental stages (*F*_24,11_ = 5.084, *p* = 0.00043, Tables S3 and S10). Expression was significantly increased during the yolk sac stages compared to Stages 6 and 9, where expression was significantly lower. Expression significantly increased again in Stages 10 and 11. In Stage 14, expression was significantly lower compared to the late larval development. The expression of *myog* differed significantly between Stage 11 and Stages 6 and 9, and Stage 14 and Stages 2, 3, 10, and 11.

The expression *mef2a* showed significant differences between the developmental stages (*F*_24,11_ = 3.456, *p* = 0.00535, Tables S3 and S11). Expression peaked in the juvenile stage, and expression was significantly lower in larval stages 3, 5, 6, and 8 compared to the juvenile stage.

The expression of *mrf4* showed significant differences between the developmental stages (*F*_24,11_ = 24.24, *p* = 2.4e^−10^, Tables S3 and S12). Expression levels during the yolk sac stages are significantly higher than in Stages 5–6 and 8–9. The expression significantly increases in Stages 10 and 11 compared to Stages 5–9. Expression peaked in the juvenile stage 14 and was significantly higher than in all larval stages.

The expression of *myh6* (Figure 3) was not significant through the larval stages (*F*_24,11_ = 1.041, *p* = 0.444, Table S3).

#### 3.2.3 Genes Involved in Skeletal or Structural Development (Figure 4)

**Figure 4:**
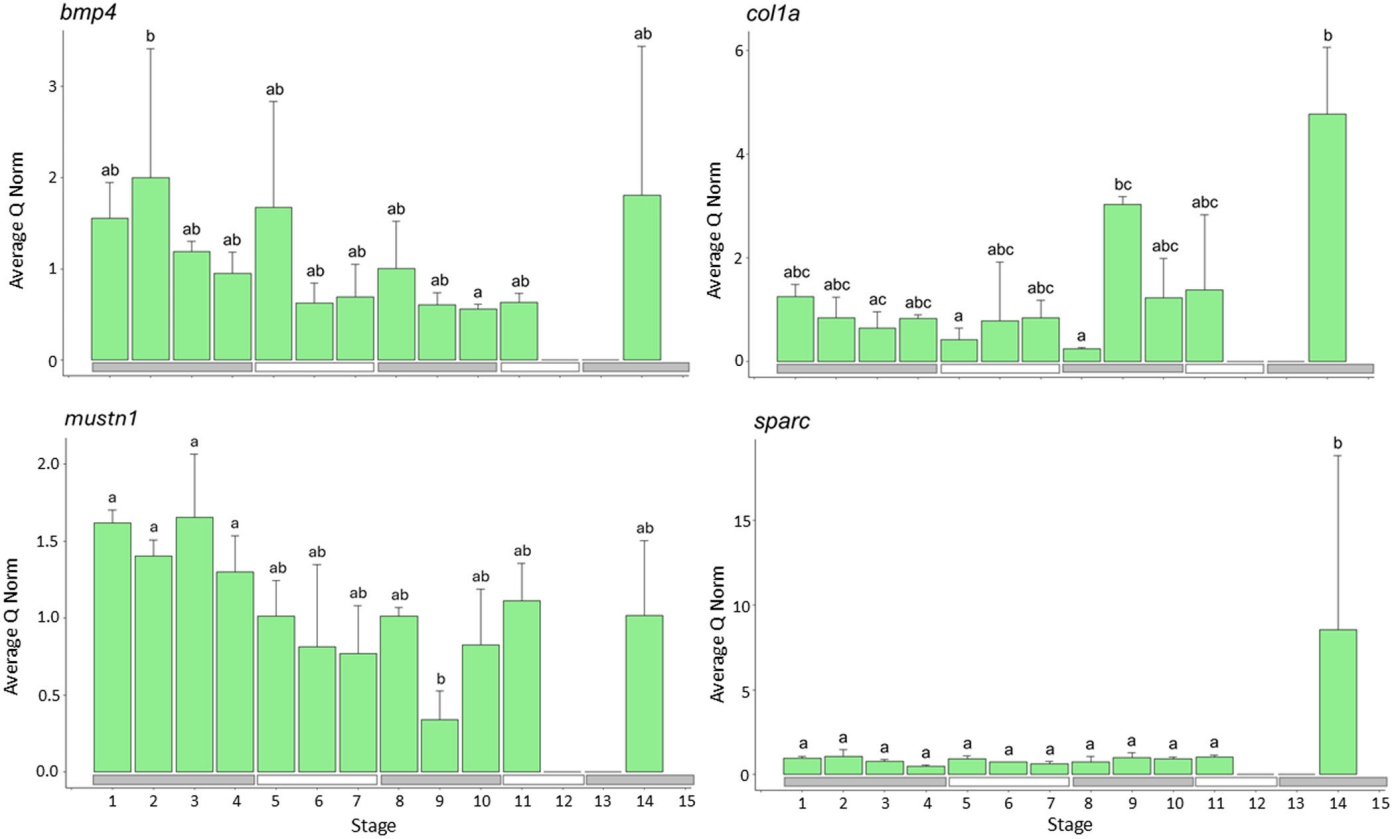
Expression of genes related to skeletal or structural development (*bmp4, col1a, mustn1, sparc*). The green bars indicate the average normed quantity of detected cDNA in *n* = 3 pools of larvae within each development stage, while the error bars indicate the positive standard deviation. The letters above the bars indicate which developmental stages do not show significant differences for *p* ≤ 0.05 (same letter) and which show significant differences (different letter). [Color figure can be viewed at wileyonlinelibrary.com]

The expression of *bmp4* differed significantly between the developmental stages (*F*_24,11_ = 3.722, *p* = 0.00344, Tables S3 and S13). Expression levels were highest at Stage 2 and differed significantly between Stages 2 and 10. Otherwise, no significant differences were found between the other stages.

The expression of *col1a* also differed significantly between the developmental stages (*F*_24,11_ = 4.842, *p* = 0.00061, Tables S3 and S14). Expression levels significantly decreased in Stages 5 and 8 compared to Stage 9 and the juvenile stage. Expression of *col1a* also differed significantly between Stage 14 and Stages 2–8.

Furthermore, the expression levels of *mustn1* differed among the developmental stages (*F*_24,11_ = 4.187, *p* = 0.00163, Tables S3 and S15). Expression in Stage 9 was significantly lower than in the yolk sac Stages 1–4.

The expression levels of *sparc* differed among the developmental stages (*F*_24,11_ = 7.751, *p* = 0.001, Tables S3 and S16). The expression of *sparc* was low during the whole larval development and increased significantly in Stage 14 of the juvenile phase.

#### 3.2.4 Genes Involved in Growth (Figure 5)

**Figure 5:**
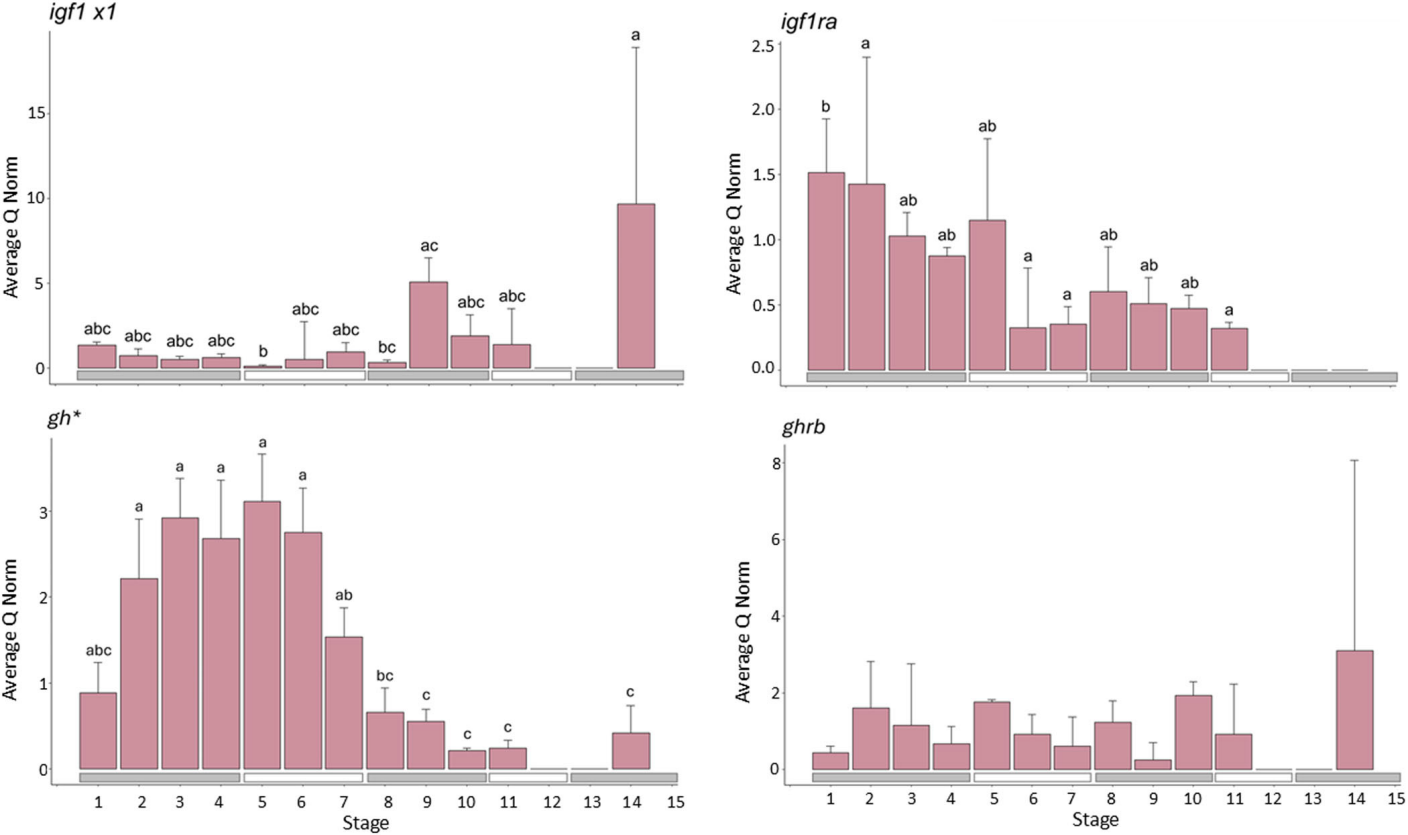
Expression of genes related to growth (*igf1 x1*, *igf1ra*, *gh*, *ghrb*). The purple bars indicate the average normed quantity of detected cDNA in *n* = 3 pools of larvae within each development stage, while the error bars indicate the positive standard deviation. The letters above the bars indicate which developmental stages do not show significant differences for *p* ≤ 0.05 (same letter) and which show significant differences (different letter). *gh* is marked with an asterisk because the gene expression measurement methods differed from the rest (see Section 2). If no difference was detected within the expression of the different stages, letters are not present. [Color figure can be viewed at wileyonlinelibrary.com]

The expression of *igf1 x1* differed significantly between the developmental stages (*F*_24,11_ = 3.878, *p* = 0.003, Tables S3 and S17). The expression of *igf1 x1* was significantly lower in Stage 5 compared to Stages 9 and 14. Expression in Stage 8 was also significantly lower compared to Stage 14.

Also, expression of *igf1ra* differed significantly among developmental stages (*F*_22,10_ = 4.813, *p* = 0.001, Tables S3 and S18). The expression of *igf1ra* differed significantly between Stages 1 and 2 and Stages 6, 7, and 11.

The expression of *gh* differed significantly between the developmental stages (*F*_24,11_ = 25.91, *p* = 0.001, Tables S3 and S19). Expression levels were significantly higher in Stages 2–6 compared to Stages 8–14.

The expression of *ghrb* did not differ significantly between the developmental stages (*F*_24,11_ = 1.179, *p* = 0.351, Table S3).

#### 3.2.5 Correlation Matrix (Figure 6)

**Figure 6:**
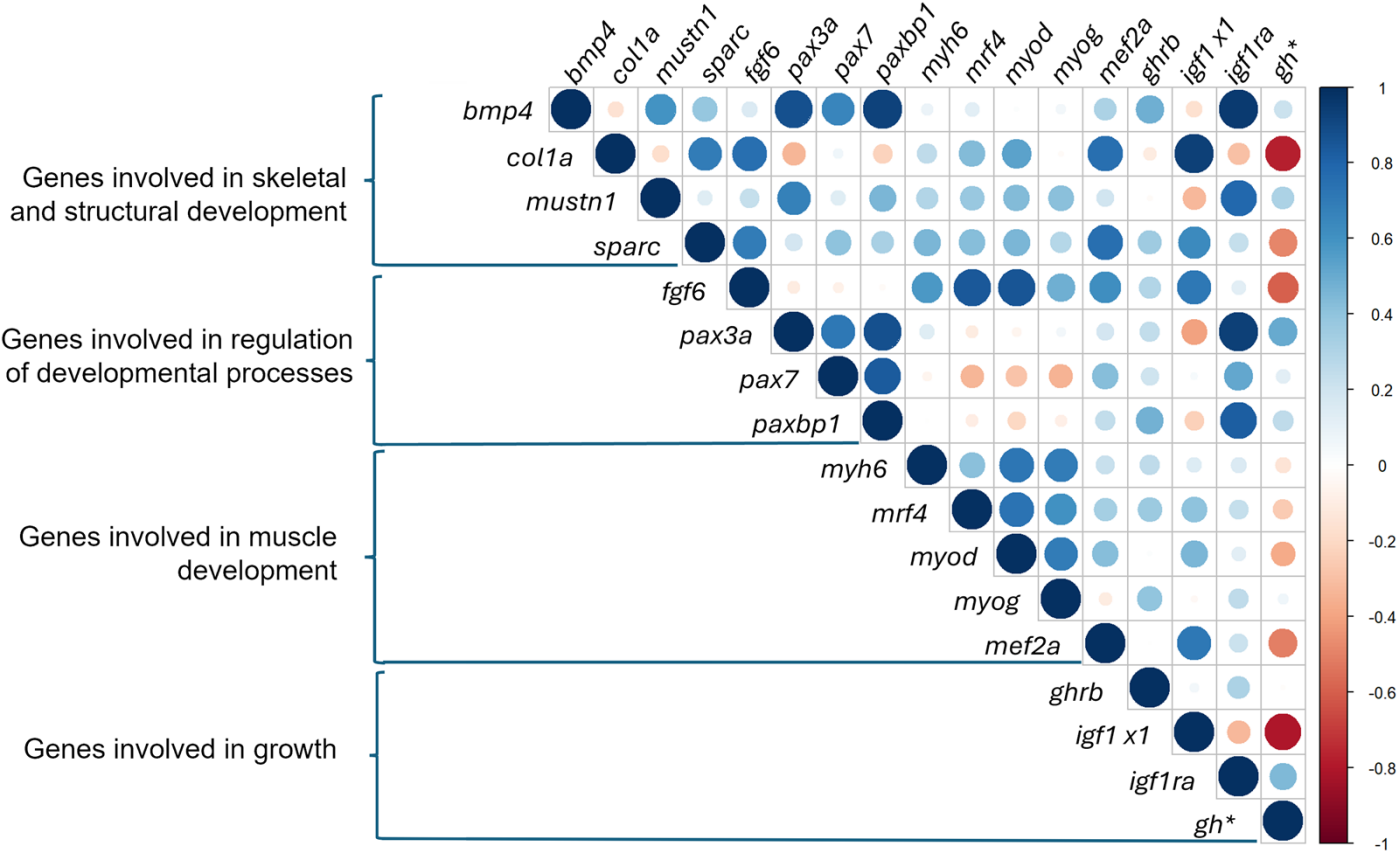
Robust correlation matrix for the expression of the different genes analyzed in this study. The blue dots indicate positive correlation, red dots indicate negative correlation. Larger and darker dots indicate stronger correlation. *gh* is marked with an asterisk because the gene expression measurement methods differed from the rest (see Section 2). [Color figure can be viewed at wileyonlinelibrary.com]

While all genes involved in the development of musculature (*myod, myog, mef2a, mrf4, myh6*) mostly showed strong positive correlations with each other, most genes involved in muscle development correlated negatively with *gh* (*myh6*, *myod*, and *mef2a*). Furthermore, *mrf4* correlated negatively with *pax7*. It is noteworthy that *mef2a* showed a slight negative correlation to *myog* but a strong positive correlation to all genes involved in developmental processes and skeletal development.

Genes involved in structural and skeletal development (*bmp4, mustn1, col1a, sparc*) correlated positively with genes involved in developmental management functions, such as *fgf6* and genes of the PAX family, except for *col1a*, which showed a negative correlation to *pax3a* and *paxbp1*.

Genes involved in growth (*gh, ghrb, igf1 x1, igf1ra*) were mostly positively correlated with genes involved in muscle development. Only *igf1 x1* was correlated negatively with *gh*, *ghrb*, and *igf1ra*, which in turn were all positively correlated with each other. Genes involved in general developmental management, such as the PAX genes, were mostly positively correlated with genes involved in growth.

### 3.3 Analysis of Histological Cross‐Sections (Figure S1)

The histological cross sections showed that in Stages 1–4, an inner and outer layer of muscle fibers was present, and over the course of the phase, the muscle cells within the inner layer mostly enlarged (not shown in Figure S1). In the subsequent Stage 5, muscle fibers elongated, and at the dorsal and ventral ridges, more muscle fibers formed. By Stage 7 the cells were partially separated by dorsal and ventral myosepta (Figure S1, Stage 8). Also, an observable increase of fibers occurred especially in Stages 10 and 11 (Figure S1). By Stage 11, the muscle fibers were organized into eight myomeres per side. From Stage 11 to Stage 14, the amount of muscle fibers visibly increased again, and additionally, red musculature developed especially along the lateral sides.

## 4 Discussion

Marine fish larvae undergo substantial developmental processes during their larval ontogeny (Miller and Kendall 2009). At hatching, skeletal, motor, and sensory systems are mostly at an early developmental stage, and most species have nonfunctional gut, gills, and/or kidneys. Thus, fundamental biological functions including locomotion, digestion, osmoregulation, and respiration exhibit distinct characteristics compared to those observed in adults (Miller and Kendall 2009). To survive, fish larvae must develop and grow rapidly while effectively managing their limited energy resources. This entails the selective prioritization of certain developmental processes over others at certain times to increase their chances of survival.

### 4.1 Alternating Growth and Developmental Prioritizing Periods During Larval Development

Previous studies on fish larvae have indicated that larger larvae generally have a higher chance of survival, largely due to their enhanced foraging efficiency and superior capacity to avoid predators (e.g., Bailey and Batty 1983; Blaxter and Batty 1985; Hubbs and Blaxter 1986; Eaton and Didomenico 1986). The length ranges observed per developmental stage in larvae sampled in 2023 are consistent with those previously established by Fischbach et al. (2023), thereby demonstrating the consistency of larval growth patterns across different years and environmental conditions. The observed length ranges suggest that herring larvae experience two key periods in which growth is prioritized. This is reflected by the high variability of body lengths in these stages. The first growth period occurs during the dorsal‐fin development phase. Stage 7 in particular stands out due to its wide range of body sizes, suggesting substantial growth variability. The second growth period takes place toward the end of larval development, where Stage 11 shows the widest size ranges of all larval stages. This pattern aligns with findings from Fischbach et al. (2023), who observed a similarly broad size range also in Stage 12. In contrast, during the yolk sac stages, growth differences between larvae are minor, with length ranges of the four stages largely overlapping. This reflects a slower and/or more uniform growth at this early stage. In the caudal fin development phase, growth slows down, as evidenced by little variable length ranges and a closer mean length between stages. Here, also, no significant difference was found between most stages. While different environmental factors, such as temperature and food availability, can certainly play a role in increasing or decreasing the variability per length range even within a season, the consistent observations from several years indicate that growth is most likely to be prioritized during the dorsal fin developmental phase and Stages 10–11.

Growth is promoted by the development of muscle tissue (Johnston 1999). Here, the expression pattern of transcription factors involved in myogenesis, such as *myod, myog, mrf4, mef2a*, also strongly supports the presence of two distinct larval myogenetic periods (Figure 3). The expression of *myod*, which initiates the muscle lineage determination (Watabe 1999; Berkes and Tapscott 2005; Francetic and Li 2011; Chen et al. 2015) and *myog*, which is involved in the terminal differentiation and formation of muscle fibers out of single myotubes (Tajbakhsh et al. 1997) is highest in Stages 10 and 11, followed by the yolk sac stages. Apparently, *myod* and *myog* expression peaks just before a major growth period. Since *myod* regulates the determination of the muscle cell lineage, which is followed by their differentiation of myotomes into myocytes and myotubes regulated by *moyg*, this process most likely must be initiated before a major myogenetic period. Also, the expression of *mef2a*, which can upregulate myogenin transcription (Johnston and Hall 2004), followed a similar pattern with higher expression during most of the yolk sac stages and Stages 9–11. Expression of both genes was lowest in the juvenile stage, potentially because most muscle cells had already terminally differentiated, and further growth mostly occurs through hypertrophy. However, interestingly, *mef2a*, shows high expression in the juvenile stage. This suggests that *mef2a*, in addition to its role in embryonic development, also possibly contributes to growth during the juvenile stages. Furthermore, this could explain the slight negative correlation between *myog* and *mef2a* (Figure 6). Larval myogenesis is involved in the increase of muscle fibers during the juvenile phase. *Mrf4* shows generally low levels of expression throughout larval development, peaking in the juvenile stage. This is consistent with previous reports that *mrf4* constitutes the last step in myogenesis, with its expression predominantly increased until the adult stage (Rhodes and Konieczny 1989). Interestingly, *mrf4* also shows slight increases in expression during the yolk sac stages and in Stages 10 and 11, similar to *myod and myog*, which complies with studies on other vertebrates suggesting that *mrf4* is expressed in a biphasic pattern: during the onset of myogenesis and in adults (Hinterberger et al. 1991).

For specific markers, such as *myh6*, which play a key role in heart muscle formation (Singleman and Holtzman 2012; Schiaffino 2018), no significant difference between the stages was found. However, expression was highest in the first two and last two stages of larval development compared to intermediate stages. This suggests that the heart muscles grew continuously throughout larval development, with higher growth occurring during both the early and final larval stages. As in herring, many organs are enlarged during the last larval stage (Joly et al. 2021); it is likely that the heart also expanded, necessitating adjustments in its muscle tissues.

Factors initiating the muscle lineage determination, such as *myod*, are commonly regulated by *pax3a* and *pax7*, which are expressed in satellite cells, with *pax3a* further activating *myod* expression (Bendall et al. 1999; Buckingham 2007; Buckingham and Relaix 2007; Koganti et al. 2020). In herring, *pax3a* expression decreased across the developmental stages, while *pax7* expression remained constant and peaked in the juvenile stage. The increase of *pax7* in the juvenile stage could indicate that juvenile growth is highly driven by satellite cells.

In fish, *fgf6* is suspected to be involved in hyperplasia, which supports the continuous growth of fish throughout their adult life due to its sustained expression (Rescan 1998). During herring development, *fgf6* expression gradually decreases and remains low until the end of the caudal fin developmental phase. Then, its level rises significantly, suggesting that *fgf6* plays a more prominent role during the second larval growth period in the late larval stages, as well as in growth during the juvenile phase.

Histological analysis of the developmental stages in this study showed that differences in muscle development first became evident at Stage 5, which corresponds to the onset of dorsal fin development. Before Stage 5, the cross‐sections of herring larvae showed large resemblances to the embryonal muscle development, described by Johnston et al. (1995), and during the yolk sac stages, it seemed that only hyperplasia of muscle cells occurred. From Stage 5 on, myoblasts aligned more orderly between myosepta and observably more muscle cells had formed, corresponding to the second muscle differentiation phase described by Johnston et al. (1998). The cross‐section also showed that in Stage 11, fewer but larger muscle fibers occurred compared to Stage 14. It is therefore possible that in Stages 12 and 13, corresponding to the third muscle differentiation phase described by Johnston et al. (1998), there is increased growth and muscle development, although a general activation of myoblasts scattered throughout the myotome can be expected.

Factors involved in growth generally showed no outstanding trends in relation to those developmental stages with large length ranges. For example, the expression of *igf1x1* was relatively low during the larval development except at the end of the caudal fin phase in Stages 9–11 and peaked in Stage 14. Since IGFS can stimulate cell proliferation, differentiation, and hypertrophy (Glass 2003, 2005), the increase in expression could give evidence that *igf1 x1* is at least partly involved in the second larval and subsequent juvenile growth period. The expression of *gh*, on the other hand, was high in the yolk sac stages and decreased during the dorsal fin development phase, and was then lowest during Stage 11. In vertebrates, GH is the main regulator of somatic growth (Björnsson et al. 2002). The expression pattern suggests that in herring, it plays a more pronounced role during the first growth period and/or by binding to *ghr*, it triggers post‐receptor signaling events which lead to the transcription of target genes such as igf1, which is then only visible in later stages.

Studies on postcranial skeletal development across different larval stages indicated that early development primarily involves the formation of skeletal elements within the fins, which first develop as cartilage. Then, from Stage 10 onwards, ossification of major skeletal elements, such as the vertebrae, occurs (Fischbach et al. 2022, 2023). The gene expression data revealed that the expression of *bmp4*, a key mediator of dorsoventral patterning in vertebrates and required for the induction of ventral fate in fish (Stickney et al. 2007) as well as intramuscular bones (Su and Dong 2018) showed a slight decrease in expression during the larval development. The same was recorded for *mustn1*, a key regulatory molecule for myogenic and chondrogenic processes (Hadjiargyrou 2018). This decline coincides with the completion of skeletal system formation by the end of the larval period. Increases in *mustn1* at the end of the larval phase could, on the other hand, coincide with increases in cartilage development, for example, at the ribs. *Col1a* encodes for collagen type 1 pro‐alpha1 chains, which are included in connective tissues, bone, tendon, and cartilage. *Col1a* is expressed throughout the whole larval period, with a significant increase in larval stage 9 and juvenile stage 14. The moderate expression levels within the yolk sac stages could be connected to chondral formations in the skull, while in Stage 11, the histological cross‐sections revealed a visible increase in myosepta and thus in connective tissue, which also contains *col1a*. *sparc* is highest expressed in Stage 14, which could be explained by its role in calcification of otoliths, bones, teeth, and scales (Renn et al. 2006; Rotllant et al. 2008; Li et al. 2009), processes that occur during the juvenile phase.

The expression of most genes tested in this study exhibited large standard deviations through all developmental stages, indicating strong individual variation in growth and myogenesis. Given that growth is largely influenced by environmental factors such as food availability and quality, temperature, and other factors, it is likely that gene expression levels differ between individuals. Contrary to this, the interspecimen variability was found to be low in pretrial tests (see Figure S2). The utilized pool numbers, based on catch numbers and methodical necessities, consequently result in a focus on developmental stage and phase comparisons. Still, no clear trend is visible between increasing standard deviation and sample size per pool. Interestingly, genes involved in skeletogenesis displayed less standard deviation, suggesting that this process is less influenced by varying environmental factors such as temperature or food availability.

### 4.2 Overview of Larval Life Stages and Potential Developmental Bottlenecks

To gain a deeper understanding of herring larval development and the overlapping developmental processes, all available information relevant to the larval stages and phases was consolidated (Table 3). Overall, in herring development, it appears that during periods of substantial growth, other processes such as skeletogenesis play a subordinate role. Consequently, periods of growth and developmental priorities alternate, resulting in the occurrence of different mortality or survival bottlenecks in accordance with the state of development (Table 4). Shortly after hatching, herring larvae rely solely on energy reserves from the yolk sac, which are gradually depleted until they are fully absorbed in Stage 4. During these early stages, the larvae have not yet developed fins, and as observed in this study, still exhibit embryonic muscle structure. Due to their presumed limited mobility, they are most likely at higher risk of drifting away during strong winds or currents. By Stage 4, herring larvae have depleted their yolk sac and are in need to find suitable prey in their immediate surroundings. In this stage, a lack of adequate food sources can lead to high mortality rates caused by starvation (Stenevik et al. 2021). This apparently creates a major bottleneck in fish development (Hjort 1914). To effectively catch adequate prey, it is essential that the larvae learn to hunt during the dorsal fin development phase. In these stages, an observable increase in both the number of muscle fibers and overall growth is evident, which is likely to enhance the larvae's mobility and, therefore, their ability to hunt and evade predators. This is in accordance with the “bigger is better” hypothesis (Bailey and Batty 1983; Blaxter and Batty 1985; Hubbs and Blaxter 1986; Eaton and Didomenico 1986). At Stage 7, the enzymatic maturation within the liver occurs, which potentially increases the larvae's ability to store energy reserves (Joly et al. 2021). It has been hypothesized that these processes may lead to high mortality rates, even when climatic and nutritional needs are evidently met (Joly et al. 2021, 2023) and thus also represents a major mortality bottleneck. Prominent changes during the caudal fin developmental phase are the flexion of the notochord and coherent skeletal processes, which potentially increase the swimming speed of the larvae (Moyano et al. 2016). Batty (1984) demonstrated that larvae also alter their swimming behavior, which apparently is coupled to the newly developed caudal fin. Therefore, from Stage 10 on, when the caudal fin is fully developed, larvae gain increased swimming abilities, resulting in a significantly increased survival rate. In the last larval phase, the pelvic fin development phase, the larvae experience another growth prioritizing period, further enhancing their swimming speed (Moyano et al. 2016). When herring begin to metamorphose into juvenile fish, they migrate from the nutrient‐dense inner‐coastal habitats to less nutrient‐dense open water environments (Polte et al. 2017), and start forming small schools. This correlates to an increase in red musculature, advancing their ability to swim constantly, in juvenile herring found in this study during Stage 14. At this time, herring are also believed to switch from cutaneous respiration to gill respiration (De Silva 1974). In accordance with this alteration in habitat, there are corresponding changes in eye morphology, with the presence of both cones and rods (Blaxter and Jones 1967). This presumably serves to adapt the visual capacities to the coastal environments. Metamorphosis also triggers retinomotor responses (Blaxter and Jones 1967; Hubbs and Blaxter 1986), and the formation of the swim bladder (Allen et al. 1976). The shift to the juvenile phase could very well constitute another bottleneck (Table 4), because many crucial developmental changes occur during this time. While for larval phases many developmental processes have already been investigated, this study also emphasizes the importance of the developmental processes occurring during the juvenile stage, which are most likely associated with a change in habitat.

**Table 3:** Overview of the developmental processes within the larval development of herring.

Stage	Morphology (Fischbach et al. 2023)	Skeletogenesis (Fischbach et al. 2022, 2023)	Myogenesis (This study)	Growth (This study)	Organogenesis (Joly et al. 2021)	Neural system (Blaxter et al. 1983)	Sensory system (Blaxter and Jones 1967)	Respiration (De Silva 1974)
1	Head is connected to the yolk sac by connective tissue, pectoral fins are immobile due to yolk sac size, eyes are fully pigmented, larval fin fold stretches around the body except for the head		Increased expression of MYOD and MYOG, muscle tissue resembles embryonic phenotype, and muscle fibers enlarge during yolk sac stage, but few new fibers are formed	Length measurements overlap strongly for all yolk sac stages and only a slight in overall length increase is visible	Short, narrow and folded esophagus lined with goblet cells, stomach appeared as simple cavity with short and mostly flattened epithelial cells, intestine lined with straight thick layer of enterocytes, some goblet cells in between, microvilli form brush border at top of the cells, elongated pancreas and liver, reach from posterior of pectoral fins to beginning of intestine, pancreatic cells organized in weakly distinct clusters, only exocrine pancreas visible, liver with large cells with large vacuoles, stores important quantities of glycogen	10 prominent neuromasts on either flank, about every fourth myotome	Fairly simple retina, no pigment movement, addition of cones by peripheral appositional growth.	Most likely cutaneous respiration
2	Head free from the yolk sac, pectoral fins are mobile, yolk sac is more depleted					Number of neuromasts on flank increases at the flanks and on the head		
3	Yolk sac is almost depleted, only remains are still visible							
4	Yolk sac is fully depleted, no remains are visible, mesenchymal condensation at the tip of notochord	Cleithrum is formed by intramembranous ossification, Coracoscapular cartilage is present			Acid mucosubstance production in the esophagus			
5	Precursor of dorsal fin is visible as a mesenchymal condensation within dorsal larval fin fold, mesenchymal condensation in the caudal fin progressed		Muscle fibers organized between ventral and dorsal myosepts, more muscle fibers form and existing fibers elongate, Growth mostly originating from distinct germinal zones of myoblast at the dorsal and ventral	Accelerated growth rates are visible through larval length measurements	Tissue organization, like last phase, acid mucosubstance production in the esophagus			
6	Pterygiophores in dorsal fin are visible	Pterygiophores in the dorsal fin form in cartilage, in caudal fin parhypural first hypural are present in cartilage						
7	Fin rays are present in dorsal fin, dorsal fin is separated from fin fold, and protrudes significantly above it, potentially fin rays in caudal fin	Ossification of fin rays			Distinction between stomachal cavity and intestine now clearly visible as structured pylorus, in the intestine two regions (mid. and hindgut) could be distinguished, endocrine pancreas was visible, enzymatic maturation of the liver			
8	Notochord flexion begins, tip of the notochord bends upwards, hypural 1 forms a visible edge, size of fin fold decreases, precursors of anal fin rays are visible			Increase in lengths per stage reduces and less growth occurs in the caudal fin stages	Organs become larger and thicker, esophagus interstice was wider and longer, clear pattern appears in mucosubstance secreation by the goblet cells of the esophagus, esophagus opens up to stomacal cavity extended in the upper part, height of epithelial cells in stomach increases and villi of the intestines become larger, endocrine portion of pancreas takes form of elongated cell cluster (islet), cells organize in distinct and circular acini, pancreas becomes more elongated, potruding beyond the beginning of the intestine, liver undergoes major changes, liver morphology is highly variable among individuals, glycogen reserves are preserved.			
9	Flexion of the notochord is complete, most posterior point of hypural 1 is almost aligned with posterior part of notochord, caudal fin is still round and shows no incision	In caudal fin, uroneural 1 forms from intramembranous ossification, cartilage precursor of basipterygium appears, posterior neural arches form in cartilage, further development in shoulder girdle						
10	Caudal fin is incised and clearly homocercal, posterior parts of the hypurals are aligned and form a straight vertical line, fin rays are present in the anal fin	Anal fin rays are ossified, parhypural and hypurals begin to ossify	Increased expression of MYOD and MYOG, more muscle fibers develop throughout the whole cross‐section and are strictly organized in myosepta, and more connective tissue has developed					
11	Pelvic fin is visible as a mesenchymal condensation with lepidotrichia precursors	Ural centrum II ossifies as chordacentra, both epurals are present in cartilage, uroneural 2, posttemporal, and supracleithrum form from intramembranous ossification, distal radials and fin stay of the anal fin develop in cartilage		Accelerated growth rates are visible through larval length measurements	The esophagus and its longitudinal folds are longer, and the intestine is wider, possible increase in the quantity of acid and mucosubstances produced, epithelial cells of stomach increase in height, ceca pyloric becomes visible, structure of the exocrine pancreas become more diffuse, pancreas expands further along the intestine			
12	Pelvic fin is fully developed with fin rays, transformation of larvae is in progress, pelvic fin is now situated less anterior to dorsal fin, body height increases	1 fuses to preural centrum I (pleurostyle), ural centrum I forms as chordacentra, neural and hemal arches ossify, spines forms, ribs develop in cartilage, radials and rays in shoulder girdle form						
Juvenile						Number of neuromasts on flank and head increase	Formation of rods	Most likely first occurrence of gills

**Table 4:** Overview of potential bottleneck stages in Atlantic herring larval development.

Developmental bottleneck	Advanced abilities	Morphological/Physiological development	Influencing abiotic/Biotic factors
Larval drift phase (Stages 1–3)			Wind/currents, predation
First feeding (Stage 4)	Possibly digestion of external food particles	Development of jaw and gut	Occurrence of (the preferred) prey organism
Larval survivors (Stage 10)	Increased swimming capability	Development of caudal fin, end of flexion phase, development of musculature	Escape predation, increased foraging capabilities
Metamorphosis to Juvenile fish (Stage 12b–Juvenile)	Transition from cutaneous respiration to respiration via gills + other transitions (eye, swim bladder), schooling behavior	Ossification of gill arches + gill rakers and opercular apparatus, change in surface and volume ratio, development of rods and swim bladder, development of red musculature	Change in habitat and mode of living

## 5 Conclusion

The present study comprises a comprehensive analysis of the developmental processes occurring in herring larvae, including myogenesis, skeletal development, and growth, across the different stages of their development. The utilization of biometric, gene expression, and histological data enabled the identification of two distinct growth periods: the first occurring during the dorsal fin developmental phase and the second taking place during the pelvic fin developmental phase. These periods were related to different myogenesis waves. By integrating these findings with existing literature on developmental processes during the larval and juvenile phases of herring, the development can be categorized into periods that prioritize specific ontogenetic processes (yolk sac and caudal fin development phase) and periods that predominantly prioritize muscle development and growth (dorsal fin and pelvic fin development phase).

## Author Contributions

**Vivian Fischbach:** conceptualization (equal), data curation (equal), formal analysis (lead), funding acquisition (supporting), investigation (lead), project administration (equal), resources (equal), validation (equal), visualization (equal), writing – original draft (lead). **George P. Franz:** conceptualization (equal), data curation (equal), formal analysis (supporting), investigation (supporting). methodology (equal), supervision (equal), validation (equal), writing – review and editing (equal). **Timo Moritz:** conceptualization (equal), funding acquisition (supporting), resources (supporting), supervision (equal), validation (supporting), visualization (equal), writing – review and editing (equal). **Daniela Ohde:** resources (equal), methodology (equal), writing – review and editing (supporting). **Philipp Thieme:** investigation (supporting), validation (supporting), writing – review and editing (equal). **Paul Kotterba:** resources (supporting), validation (supporting), writing – review and editing (supporting). **Patrick Polte:** resources (equal), supervision (supporting), validation (supporting), writing – review and editing (supporting). **Bianka Grunow:** conceptualization (equal), validation (supporting), funding acquisition (lead), methodology (equal), project administration (lead), resources (lead), supervision (equal), writing – review and editing (equal).

## Supporting information


**Figure 1:** HE stained Histological cross sections of herring larvae in stages 8‐11 and 14. The arrows mark important structures such as the gut, notochord, red and white muscle, myosepta, fin rays & pterygiophores and spinal cord and chorda. **Figure 2:** Expression of genes related to general developmental processes (*fgf6, pay3a, pax7 & paxbp1*), muscle development (*myod, myog, mef2a, mrf4, myh6*),

## Data Availability

Raw data (sequencing data and gene expression output and normlaized data) as well as corresponding metadata will be published under https://doi.org/10.5281/zenodo.14621189 and for peer review purposes can be accessed via: https://zenodo.org/records/14621189?preview=1&token=eyJhbGciOiJIUzUxMiJ9.eyJpZCI6IjE4YmRmM2EyLTAzOTQtNDQ1ZS04ZTA5LWFlNTU2OWJmNjI3ZiIsImRhdGEiOnt9LCJyYW5kb20iOiJkZmRiNDMxZjM4ZmI3NTY0NGE5ZWU2Y2JkMTllMzVhYSJ9.4fRGV-NSmXK3sj2NJt_rP03NZDdGa-XY4bTKr-fy9eGwFmC6zQXXx_BkJWiJVHjA70JIqK3iv7B2aUI7dsrT3Q.
