# Supplementary material for: Growth and Developmental Processes Alternate During Larval Development of Atlantic Herring
Source: Evol Dev. 2025 Nov 22;27(4):e70022. doi: 10.1111/ede.70022 (PMC12639411; doi:10.1111/ede.70022)
Supplement: Supplementary file 1 — Figure 1: HE stained Histological cross sections of herring larvae in stages 8‐11 and 14. The arrows mark important structures such as the gut, notochord, red and white muscle, myosepta, fin rays & pterygiophores and spinal cord and chorda. Figure 2: Expression of genes related to general developmental processes (fgf6, pay3a, pax7 & paxbp1), muscle development (myod, myog, mef2a, mrf4, myh6), [file EDE-27-e70022-s001.docx]

**Supplementary Tables and Figures:**

Tab. 1: Overview of number of larvae per stage measured for length ranges per stage within station 207 (Lat:54.276350, Lon:13.188083) of the RHLS.

| Stage | 1 | 2 | 3 | 4 | 5 | 6 | 7 | 8 | 9 | 10 | 11 |
| --- | --- | --- | --- | --- | --- | --- | --- | --- | --- | --- | --- |
| Cruise |  |  |  |  |  |  |  |  |  |  |  |
| 1 |  |  |  | 1 |  |  |  |  |  |  |  |
| 2 |  | 2 | 1 | 2 |  |  |  |  |  |  |  |
| 3 | 2 | 1 |  | 2 |  |  |  |  |  |  |  |
| 4 | 3 | 3 | 1 | 3 |  |  |  |  |  |  |  |
| 5 | 5 | 30 | 30 | 11 |  |  |  |  |  |  |  |
| 6 | 3 | 30 | 30 | 15 |  |  |  |  |  |  |  |
| 7 |  | 13 | 20 | 30 |  |  |  |  |  |  |  |
| 8 | 5 | 9 | 8 | 30 | 14 |  |  |  |  |  |  |
| 9 |  |  |  | 30 | 24 | 10 | 1 | 2 |  |  |  |
| 10 |  | 1 |  | 27 | 26 | 24 | 30 |  |  |  |  |
| 11 | 3 | 14 | 10 | 20 | 12 | 28 | 30 | 2 | 1 |  |  |
| 12 |  |  | 1 | 30 | 30 | 23 | 19 |  | 1 |  |  |
| 13 |  | 12 | 3 | 30 | 11 | 23 | 30 |  |  |  | 1 |
| 14 |  |  |  | 20 | 21 | 14 | 30 | 30 | 30 | 30 | 15 |
| 15 |  |  |  |  | 2 | 19 | 21 | 9 | 9 | 13 | 12 |
| 16 |  |  |  |  |  |  | 1 | 1 | 2 |  |  |
| 17 |  |  |  |  |  | 1 |  |  |  |  | 1 |

Tab. 2: Dehydration protocols for smaller and larger larvae.

| Steps | Protocol Stage 1-6 | Protocol Stages 7-14 |
| --- | --- | --- |
| 70% Isopropanol | 5 min | 1 h |
| 70% Isopropanol | 5 min | 1h |
| 80% Isopropanol | 5 min | 1h |
| 80% Isopropanol | 5 min | 1h |
| 96% Isopropanol | 5 min | 1h |
| 100% Isopropanol | 10 min | 1h |
| 100% Isopropanol | 10 min | 1h |
| Roticlear | 10 min | 1h |
| Roticlear | 10 min | 1h |
| Paraffin | 30 min | 1h |
| Paraffin | 1h | 1h |
| Paraffin | 1h | 1h |
| Paraffin | 1h | 1h |

Tab. 3: p-values of Shapiro, Kruskal Wallis and ANOVA.

| Gene | Shapiro | Skewness | Kruskal Wallis | Shapiro (trans. data) | Levene’s  test | ANOVA |
| --- | --- | --- | --- | --- | --- | --- |
| Length ranges | < 2.2e-16 | 5.659273 | < 2.2e-16 | < 2.2e-16 | 2.039e-07 | F_1075,10_= 835.1, *p* = <2e-16 |
| *fgf6* | 3.393e-05 | 1.827966 | 0.0007518 | 0.5997 | 0.6199 | F_24,11_= 12.63, *p* = 1.85e-07 |
| *pax3a* | 0.00064 | 1.562614 | 0.01602 | 0.2948 | 0.007442 | F_23,11_= 2.571, *p* = 0.0271 |
| *pax7* | 6.033e-09 | 3.919679 | 0.06532 | 0.006453 | 0.3055 | F_24,11_= 2.324, *p* = 0.0408 |
| *paxbp1* | 0.000698 | 1.403346 | 0.06987 | 0.3996 | 0.7677 | F_23,11_= 2.64, *p* = 0.0239 |
| *myod* | 1.593e-05 | 1.549869 | 0.01102 | 0.08029 | 0.1225 | F_23,11_= 6.83, *p* = 5.58e-05 |
| *myog* | 0.2643 | 0.3453823 | 0.01355 | - | 0.973 | F_24,11_= 5.084, *p* = 0.00043 |
| *mef2a* | 1.901e-05 | 1.540802 | 0.04757 | 0.6472 | 0.8677 | F_24,11_= 3.456, *p* = 0.00535 |
| *mrf4* | 8.392e-10 | 2.862761 | 0.001038 | 0.03165 | 0.3115 | F_24,11_= 24.24, *p* = 2.4e-10 |
| *myh6* | 4.403e-06 | 2.542097 | 0.02867 | 0.01995 | 0.02952 | F_24,11_= 1.041, *p* = 0.444 |
| *bmp4* | 1.588e-06 | 2.052822 | 0.009265 | 0.1927 | 0.7246 | F_24,11_= 3.722, *p* = 0.00344 |
| *col1a* | 1.397e-05 | 1.650837 | 0.01402 | 0.8712 | 0.7176 | F_24,11_= 4.842, *p* = 0.00061 |
| *mustn1* | 0.5617 | -0.0917 | 0.01774 | - | 0.9462 | F_24,11_= 4.187, *p* = 0.00163 |
| *sparc* | 7.089e-12 | 4.076849 | 0.01511 | 2.315e-08 | 0.4267 | F_24,11_= 7.751, *p* = 1.56e-05 |
| *igf1 x1* | 2.494e-09 | 3.325881 | 0.007438 | 0.7849 | 0.5671 | F_24,11_= 3.878, *p* = 0.00266 |
| *Igf1ra* | 0.002004 | 1.335064 | 0.01602 | 0.9316 | 0.838 | F_22,10_= 4.813, *p* = 0.00103 |
| *gh* | 0.001271 | 0.3600728 | 0.0009505 | 0.00511 | 0.9668 | F_24,11_= 25.91, *p* = 1.18e-10 |
| *ghrb* | 8.294e-08 | 2.888073 | 0.3097 | 0.1848 | 0.885 | F_24,11_= 1.179, *p* = 0.351 |

| **Stage** | **1** | **2** | **3** | **4** | **5** | **6** | **7** | **8** | **9** | **10** | **11** |
| --- | --- | --- | --- | --- | --- | --- | --- | --- | --- | --- | --- |
| **1** |  | 1 | 1 | 2.44e- 2 | 1.83e- 9 | 2.91e-17 | 1.70e-26 | 4.08e-25 | 9.70e-28 | 2.14e-30 | 2.15e-28 |
| **2** | n.s. |  | 1 | 4.16e- 2 | 2.46e-17 | 1.69e-37 | 1.82e-65 | 3.09e-40 | 8.54e-45 | 9.18e-50 | 8.33e-41 |
| **3** | n.s. | n.s. |  | 1 | 2.15e- 9 | 4.11e-25 | 7.21e-48 | 5.54e-31 | 4.66e-35 | 1.92e-39 | 9.63e-33 |
| **4** | * | * | ns |  | 4.69e-11 | 1.31e-32 | 1.13e-66 | 5.53e-34 | 1.42e-38 | 1.26e-43 | 1.19e-34 |
| **5** | *** | *** | *** | *** |  | 4.63e- 4 | 1.76e-16 | 4.00e-12 | 5.42e-15 | 4.72e-18 | 2.20e-15 |
| **6** | *** | *** | *** | *** | *** |  | 2.27e- 3 | 4.98e- 4 | 6.72e- 6 | 6.21e- 8 | 3.40e- 7 |
| **7** | *** | *** | *** | *** | *** | ** |  | 1 | 1 | 2.96e- 2 | 2.20e- 2 |
| **8** | *** | *** | *** | *** | *** | *** | n.s. |  | 1 | 1 | 1 |
| **9** | *** | *** | *** | *** | *** | *** | n.s. | n.s. |  | 1 | 1 |
| **10** | *** | *** | *** | *** | *** | *** | * | n.s. | n.s. |  | 1 |
| **11** | *** | *** | *** | *** | *** | *** | * | n.s. | n.s. | n.s. |  |

Tab. 4: p-adj. values Kruskal Walls comparison length per stage ranges. N.s. signifies p > 0.05 ; * = p ≤ 0.05 ; ** = p ≤ 0.01 ; *** = p ≤ 0.001.

Tab. 5: p-adj.-values ANOVA comparison expression levels of *fgf6* between developmental stages. N.s. signifies p > 0.05 ; * = p ≤ 0.05 ; ** = p ≤ 0.01 ; *** = p ≤ 0.001.

| **Stage** | **1** | **2** | **3** | **4** | **5** | **6** | **7** | **8** | **9** | **10** | **11** | **14** |
| --- | --- | --- | --- | --- | --- | --- | --- | --- | --- | --- | --- | --- |
| **1** |  | 0.9999271 | 0.9532073 | 0.1574336 | 0.0163237 | 0.0035493 | 0.1344658 | 0.0019348 | 0.5334557 | 0.3453006 | 0.8481411 | 0.9999454 |
| **2** | n.s. |  | 0.9997189 | 0.4484073 | 0.0685385 | 0.0163951 | 0.4002802 | 0.0091099 | 0.8927828 | 0.1103041 | 0.4676746 | 0.9741963 |
| **3** | n.s. | n.s. |  | 0.8764656 | 0.2793154 | 0.0842490 | 0.8398702 | 0.0495494 | 0.9990444 | 0.0222742 | 0.1391403 | 0.6729690 |
| **4** | n.s. | n.s. | n.s. |  | 0.9934571 | 0.8444249 | 1 | 0.7086617 | 0.9995334 | 0.0004481 | 0.0036188 | 0.0436225 |
| **5** | * | n.s. | n.s. | n.s. |  | 0.9999291 | 0.9966564 | 0.9984647 | 0.7725903 | 0.0000353 | 0.0002760 | 0.0036963 |
| **6** | ** | * | n.s. | n.s. | n.s. |  | 0.8804198 | 1 | 0.3825913 | 0.0000080 | 0.0000591 | 0.0007771 |
| **7** | n.s |  | n.s. | n.s. | n.s. | n.s. |  | 0.7572034 | 0.9988355 | 0.0003665 | 0.0029627 | 0.0363326 |
| **8** | ** | ** | * | n.s. | n.s. | n.s. | n.s. |  | 0.2587516 | 0.0000045 | 0.0000327 | 0.0004227 |
| **9** | n.s. | n.s. | n.s. | n.s. | n.s. | n.s. | n.s. | n.s. |  | 0.0030015 | 0.0229727 | 0.2096849 |
| **10** | n.s. | n.s. | * | *** | *** | *** | *** | ** | ** |  | 0.9988986 | 0.7230321 |
| **11** | n.s. | n.s. | n.s. | * | *** | *** | ** | *** | * | n.s. |  | 0.9930081 |
| **14** | n.s. | n.s. | n.s. | * | ** | *** | * | * | n.s. | n.s. | n.s. |  |

Tab. 6: p-adj.-values of ANOVA to compare expression levels of *pax3a* between developmental stages. N.s. signifies p > 0.05 ; * = p ≤ 0.05 ; ** = p ≤ 0.01 ; *** = p ≤ 0.001.

| **Stage** | **1** | **2** | **3** | **4** | **5** | **6** | **7** | **8** | **9** | **10** | **11** | **14** |
| --- | --- | --- | --- | --- | --- | --- | --- | --- | --- | --- | --- | --- |
| **1** |  | 1 | 0.9994861 | 0.9935855 | 1 | 0.9626103 | 0.3147263 | 0.9052971 | 0.7484583 | 0.2065392 | 0.0939364 | 0.9977377 |
| **2** | n.s. |  | 0.9964818 | 0.9770288 | 0.9999990 | 0.9129324 | 0.2299862 | 0.8238805 | 0.6321141 | 0.1455770 | 0.0633267 | 0.9908683 |
| **3** | n.s. | n.s. |  | 1 | 0.9999814 | 0.9999848 | 0.7792004 | 0.9996225 | 0.9913238 | 0.6276509 | 0.3756739 | 1 |
| **4** | n.s. | n.s. | n.s. |  | 0.9992161 | 1 | 0.9011627 | 0.9999945 | 0.9991858 | 0.7858093 | 0.5328013 | 1 |
| **5** | n.s. | n.s. | n.s. | n.s. |  | 0.9904993 | 0.4384244 | 0.9652894 | 0.8646807 | 0.3026135 | 0.1467081 | 0.9997620 |
| **6** | n.s. | n.s. | n.s. | n.s. | n.s. |  | 0.9722042 | 1 | 0.9999852 | 0.9110632 | 0.7089711 | 1 |
| **7** | n.s. | n.s. | n.s. | n.s. | n.s. | n.s. |  | 0.9929940 | 0.9997379 | 1 | 0.9998889 | 0.9468955 |
| **8** | n.s. | n.s. | n.s. | n.s. | n.s. | n.s. | n.s. |  | 0.9999999 | 0.9656766 | 0.8269694 | 0.9999977 |
| **9** | n.s. | n.s. | n.s. | n.s. | n.s. | n.s. | n.s. | n.s. |  | 0.9962624 | 0.9483501 | 0.9996603 |
| **10** | n.s. | n.s. | n.s. | n.s. | n.s. | n.s. | n.s. | n.s. | n.s. |  | 0.9999991 | 0.8721593 |
| **11** | n.s. | n.s. | n.s. | n.s. | n.s. | n.s. | n.s. | n.s. | n.s. | n.s. |  | 0.6730629 |
| **14** | n.s. | n.s. | n.s. | n.s. | n.s. | n.s. | n.s. | n.s. | n.s. | n.s. | n.s. |  |

Tab. 7: p-adj.-values of ANOVA to compare expression levels of *pax7* between developmental stages. N.s. signifies p > 0.05 ; * = p ≤ 0.05 ; ** = p ≤ 0.01 ; *** = p ≤ 0.001.

| **Stage** | **1** | **2** | **3** | **4** | **5** | **6** | **7** | **8** | **9** | **10** | **11** | **14** |
| --- | --- | --- | --- | --- | --- | --- | --- | --- | --- | --- | --- | --- |
| **1** |  | 1 | 0.9995718 | 0.9696087 | 1 | 1 | 0.4381349 | 0.9989474 | 1 | 0.9238731 | 0.9116340 | 0.7937933 |
| **2** | n.s. |  | 0.9999848 | 0.9926940 | 0.9999999 | 1 | 0.5773583 | 0.9999409 | 1 | 0.9738858 | 0.9679058 | 0.6620248 |
| **3** | n.s. | n.s. |  | 0.9999907 | 0.9985522 | 0.9999909 | 0.8832545 | 1 | 0.9999254 | 0.9997821 | 0.9996462 | 0.3325527 |
| **4** | n.s. | n.s. | n.s. |  | 0.9472959 | 0.9940659 | 0.9919263 | 0.9999983 | 0.9858336 | 1 | 1 | 0.1329829 |
| **5** | n.s. | n.s. | n.s. | n.s. |  | 0.9999998 | 0.3746649 | 0.9969335 | 1 | 0.8847795 | 0.8691816 | 0.8491385 |
| **6** | n.s. | n.s. | n.s. | n.s. | n.s. |  | 0.5947400 | 0.9999619 | 1 | 0.9776442 | 0.9722944 | 0.6448885 |
| **7** | n.s. | n.s. | n.s. | n.s. | n.s. | n.s. |  | 0.9126461 | 0.5171499 | 0.9984958 | 0.9989802 | 0.0124316 |
| **8** | n.s. | n.s. | n.s. | n.s. | n.s. | n.s. | n.s. |  | 0.9997693 | 0.9999302 | 0.9998776 | 0.2931534 |
| **9** | n.s. | n.s. | n.s. | n.s. | n.s. | n.s. | n.s. | n.s. |  | 0.9571115 | 0.9486708 | 0.7203358 |
| **10** | n.s. | n.s. | n.s. | n.s. | n.s. | n.s. | n.s. | n.s. | n.s. |  | 1 | 0.0897575 |
| **11** | n.s. | n.s. | n.s. | n.s. | n.s. | n.s. | n.s. | n.s. | n.s. | n.s. |  | 0.0830006 |
| **14** | n.s. | n.s. | n.s. | n.s. | n.s. | n.s. | * | n.s. | n.s. | n.s. | n.s. |  |

Tab. 8: p-adj.-values of ANOVA to compare expression levels of *paxbp1* between developmental stages. N.s. signifies p > 0.05 ; * = p ≤ 0.05 ; ** = p ≤ 0.01 ; *** = p ≤ 0.001.

| **Stage** | **1** | **2** | **3** | **4** | **5** | **6** | **7** | **8** | **9** | **10** | **11** | **14** |
| --- | --- | --- | --- | --- | --- | --- | --- | --- | --- | --- | --- | --- |
| **1** |  | 0.999042 | 1 | 0.98916 | 0.964821 | 0.9998345 | 0.283629 | 0.9999997 | 0.9974087 | 0.7913921 | 0.8379962 | 1 |
| **2** | n.s. |  | 0.988259 | 0.69675 | 0.999996 | 0.9080558 | 0.057291 | 0.9799572 | 0.8000332 | 0.2987571 | 0.3445252 | 0.9999965 |
| **3** | n.s. | n.s. |  | 0.99915 | 0.879530 | 0.9999993 | 0.438372 | 1 | 0.9999164 | 0.9184797 | 0.9446553 | 0.9999860 |
| **4** | n.s. | n.s. | n.s. |  | 0.401975 | 0.9999988 | 0.903549 | 0.9996848 | 1 | 0.9998741 | 0.9999700 | 0.9709698 |
| **5** | n.s. | n.s. | n.s. | n.s. |  | 0.6644936 | 0.019936 | 0.8427312 | 0.5096503 | 0.1278840 | 0.1523983 | 0.9982343 |
| **6** | n.s. | n.s. | n.s. | n.s. | n.s. |  | 0.688909 | 0.9999999 | 1 | 0.9905464 | 0.9953062 | 0.9982175 |
| **7** | n.s. | n.s. | n.s. | n.s. | * |  |  | 0.4889702 | 0.8266395 | 0.9986579 | 0.9968056 | 0.2849225 |
| **8** | n.s. | n.s. | n.s. | n.s. | n.s. | n.s. | n.s. |  | 0.9999788 | 0.9426526 | 0.9629702 | 0.9999489 |
| **9** | n.s. | n.s. | n.s. | n.s. | n.s. | n.s. | n.s. | n.s. |  | 0.9988626 | 0.9995961 | 0.9892166 |
| **10** | n.s. | n.s. | n.s. | n.s. | n.s. | n.s. | n.s. | n.s. | n.s. |  | 1 | 0.7414091 |
| **11** | n.s. | n.s. | n.s. | n.s. | n.s. | n.s. | n.s. | n.s. | n.s. | n.s. |  | 0.7875955 |
| **14** | n.s. | n.s. | n.s. | n.s. | n.s. | n.s. | n.s. | n.s. | n.s. | n.s. | n.s. |  |

Tab. 9: p-adj.-values of ANOVA to compare expression levels of *myod* between developmental stages. N.s. signifies p > 0.05 ; * = p ≤ 0.05 ; ** = p ≤ 0.01 ; *** = p ≤ 0.001.

| **Stage** | **1** | **2** | **3** | **4** | **5** | **6** | **7** | **8** | **9** | **10** | **11** | **14** |
| --- | --- | --- | --- | --- | --- | --- | --- | --- | --- | --- | --- | --- |
| **1** |  | 0.999931 | 0.9999645 | 0.9132569 | 0.2308318 | 0.2164390 | 0.8533646 | 0.0469005 | 0.7786856 | 0.7055111 | 0.4059144 | 0.9785621 |
| **2** | n.s. |  | 1 | 0.9984704 | 0.5731191 | 0.5495875 | 0.9941472 | 0.1689195 | 0.9834324 | 0.3259331 | 0.1402619 | 0.9998990 |
| **3** | n.s. | n.s. |  | 0.9977337 | 0.5472342 | 0.5238691 | 0.9920186 | 0.1565679 | 0.9787593 | 0.3468843 | 0.1515566 | 0.9998303 |
| **4** | n.s. | n.s. | n.s. |  | 0.9723817 | 0.9662896 | 1 | 0.6210284 | 1 | 0.0626947 | 0.0215891 | 1 |
| **5** | n.s. | n.s. | n.s. | n.s. |  | 1 | 0.9891982 | 0.9993116 | 0.9965739 | 0.0034405 | 0.0010804 | 0.9728493 |
| **6** | n.s. | n.s. | n.s. | n.s. | n.s. |  | 0.9861390 | 0.9995530 | 0.9953174 | 0.0031495 | 0.0009887 | 0.9675077 |
| **7** | n.s. | n.s. | n.s. | n.s. | n.s. | n.s. |  | 0.7152521 | 1 | 0.0452865 | 0.0152576 | 1 |
| **8** | n.s. | * | n.s. | n.s. | n.s. | n.s. | n.s. |  | 0.7993024 | 0.0004987 | 0.0001581 | 0.6765843 |
| **9** | n.s. | n.s. | n.s. | n.s. | n.s. | n.s. | n.s. | n.s. |  | 0.0327035 | 0.0108238 | 0.9999994 |
| **10** | n.s. | n.s. | n.s. | n.s. | ** | ** | * | ** | * |  | 0.9999954 | 0.1684385 |
| **11** | n.s. | n.s. | n.s. | * | *** | *** | * | *** | * | n.s. |  | 0.0715304 |
| **14** | n.s. | n.s. | n.s. | n.s. | n.s. | n.s. | n.s. | n.s. | n.s. | n.s. | n.s. |  |

Tab. 10: p-adj.-values of ANOVA to compare expression levels of *myog* between developmental stages. N.s. signifies p > 0.05 ; * = p ≤ 0.05 ; ** = p ≤ 0.01 ; *** = p ≤ 0.001.

| **Stage** | **1** | **2** | **3** | **4** | **5** | **6** | **7** | **8** | **9** | **10** | **11** | **14** |
| --- | --- | --- | --- | --- | --- | --- | --- | --- | --- | --- | --- | --- |
| **1** |  | 0.914383 | 0.5811045 | 1 | 0.9996434 | 0.9949804 | 0.9999919 | 0.9999993 | 0.9981174 | 0.6389958 | 0.1158072 | 0.5497190 |
| **2** | n.s. |  | 0.9999415 | 0.8247973 | 0.9996154 | 0.3460317 | 0.6524786 | 0.7180274 | 0.4074006 | 0.9999878 | 0.8690029 | 0.0355221 |
| **3** | n.s. | n.s. |  | 0.4469061 | 0.9507633 | 0.1138092 | 0.2863286 | 0.3386858 | 0.1415980 | 1 | 0.9953241 | 0.0082890 |
| **4** | n.s. | n.s. | n.s. |  | 0.9966711 | 0.9993619 | 1 | 1 | 0.9998423 | 0.5024814 | 0.0745745 | 0.6859582 |
| **5** | n.s. | n.s. | n.s. | n.s. |  | 0.8028593 | 0.9730746 | 0.9857779 | 0.8571873 | 0.9687317 | 0.4230506 | 0.1721819 |
| **6** | n.s. | n.s. | n.s. | n.s. | n.s. |  | 0.8028593 | 0.9730746 | 0.9857779 | 0.8571873 | 0.9687317 | 0.4230506 |
| **7** | n.s. | n.s. | n.s. | n.s. | n.s. | n.s. |  | 1 | 0.9999995 | 0.3307561 | 0.0390081 | 0.8504269 |
| **8** | n.s. | n.s. | n.s. | n.s. | n.s. | n.s. | n.s. |  | 0.9999938 | 0.3877217 | 0.0493075 | 0.7977542 |
| **9** | n.s. | n.s. | n.s. | n.s. | n.s. | n.s. | n.s. | n.s. |  | 0.1682063 | 0.0158320 | 0.9707752 |
| **10** | n.s. | n.s. | n.s. | n.s. | n.s. | n.s. | n.s. | n.s. | n.s. |  | 0.9906511 | 0.0102903 |
| **11** | n.s. | n.s. | n.s. | n.s. | n.s. | n.s. | n.s. | * | * | n.s. |  | 0.0007089 |
| **14** | n.s. | * | ** | n.s. | n.s. | n.s. | n.s. | n.s. | n.s. | * | *** |  |

Tab. 11: p-adj.-values of ANOVA to compare expression levels of *mef2a* between developmental stages. N.s. signifies p > 0.05 ; * = p ≤ 0.05 ; ** = p ≤ 0.01 ; *** = p ≤ 0.001.

| **Stage** | **1** | **2** | **3** | **4** | **5** | **6** | **7** | **8** | **9** | **10** | **11** | **14** |
| --- | --- | --- | --- | --- | --- | --- | --- | --- | --- | --- | --- | --- |
| **1** |  | 1 | 0.9441583 | 0.9992590 | 0.7870682 | 0.9540185 | 0.9906385 | 0.1672439 | 0.9999838 | 0.9999545 | 0.9998316 | 0.5321631 |
| **2** | n.s. |  | 0.9563148 | 0.9995979 | 0.8147570 | 0.9645773 | 0.9936567 | 0.1843182 | 0.9999598 | 0.9999815 | 0.9999210 | 0.4991731 |
| **3** | n.s. | n.s. |  | 0.9999633 | 0.9999995 | 1 | 1 | 0.9036089 | 0.6935827 | 0.9993595 | 0.9997821 | 0.0419405 |
| **4** | n.s. | n.s. | n.s. |  | 0.9961718 | 0.9999822 | 1 | 0.5795095 | 0.9551452 | 1 | 1 | 0.1452702 |
| **5** | n.s. | n.s. | n.s. | n.s. |  | 0.9999987 | 0.9998199 | 0.9861690 | 0.4489226 | 0.9826529 | 0.9903041 | 0.0172743 |
| **6** | n.s. | n.s. | n.s. | n.s. | n.s. |  | 1 | 0.8879282 | 0.7190472 | 0.9996091 | 0.9998776 | 0.0460043 |
| **7** | n.s. | n.s. | n.s. | n.s. | n.s. | n.s. |  | 0.7551051 | 0.8624485 | 0.9999949 | 0.9999993 | 0.0822859 |
| **8** | n.s. | n.s. | n.s. | n.s. | n.s. | n.s. | n.s. |  | 0.0547328 | 0.4496768 | 0.5035282 | 0.0010559 |
| **9** | n.s. | n.s. | n.s. | n.s. | n.s. | n.s. | n.s. | n.s. |  | 0.9860550 | 0.9761309 | 0.8543426 |
| **10** | n.s. | n.s. | n.s. | n.s. | n.s. | n.s. | n.s. | n.s. | n.s. |  | 1 | 0.2132245 |
| **11** | n.s. | n.s. | n.s. | n.s. | n.s. | n.s. | n.s. | n.s. | n.s. | n.s. |  | 0.1819717 |
| **14** | n.s. | n.s. | * | n.s. | ** | ** | n.s. | *** | n.s. | n.s. | n.s. |  |

Tab. 12: p-adj.-values of ANOVA to compare expression levels of *mrf4* between developmental stages. N.s. signifies p > 0.05 ; * = p ≤ 0.05 ; ** = p ≤ 0.01 ; *** = p ≤ 0.001.

| **Stage** | **1** | **2** | **3** | **4** | **5** | **6** | **7** | **8** | **9** | **10** | **11** | **14** |
| --- | --- | --- | --- | --- | --- | --- | --- | --- | --- | --- | --- | --- |
| **1** |  | 0.9357871 | 0.9336403 | 0.9754972 | 0.2358445 | 0.1205885 | 0.9427186 | 0.1281436 | 0.1010530 | 0.5036692 | 0.1455173 | 0.0000005 |
| **2** | n.s. |  | 1 | 1 | 0.0109301 | 0.0045995 | 0.1994586 | 0.0049561 | 0.0037137 | 0.9992534 | 0.8879987 | 0.0000132 |
| **3** | n.s. | n.s. |  | 1 | 0.0107323 | 0.0045146 | 0.1967094 | 0.0048647 | 0.0036449 | 0.9993150 | 0.8909843 | 0.0000134 |
| **4** | n.s. | n.s. | n.s. |  | 0.0172558 | 0.0073437 | 0.2787633 | 0.0079072 | 0.0059406 | 0.9952466 | 0.7979030 | 0.0000084 |
| **5** | n.s. | * | * | * |  | 0.9999998 | 0.9585418 | 0.9999999 | 0.9999980 | 0.0015085 | 0.0002288 | 0.0000000 |
| **6** | n.s. | ** | ** | ** | n.s. |  | 0.8342870 | 1 | 1 | 0.0006233 | 0.0000955 | 0.0000000 |
| **7** | n.s. | n.s. | n.s. | n.s. | n.s. | n.s. |  | 0.8488810 | 0.7890073 | 0.0374422 | 0.0061828 | 0.0000000 |
| **8** | n.s. | ** | ** | ** | n.s. | n.s. | n.s. |  | 1 | 0.0006722 | 0.0001029 | 0.0000000 |
| **9** | n.s. | ** | ** | ** | n.s. | n.s. | n.s. | n.s. |  | 0.0005023 | 0.0000773 | 0.0000000 |
| **10** | n.s. | n.s. | n.s. | n.s. | ** | ** | * | *** | n.s. |  | 0.9995659 | 0.0000916 |
| **11** | n.s. | n.s. | n.s. | n.s. | *** | *** | ** | *** | *** | n.s. |  | 0.0005973 |
| **14** | ***. | *** | *** | *** | *** | *** | *** | *** | *** | *** | *** |  |

Tab. 13: p-adj.-values of ANOVA to compare expression levels of *bmp4* between developmental stages. N.s. signifies p > 0.05 ; * = p ≤ 0.05 ; ** = p ≤ 0.01 ; *** = p ≤ 0.001.

| **Stage** | **1** | **2** | **3** | **4** | **5** | **6** | **7** | **8** | **9** | **10** | **11** | **14** |
| --- | --- | --- | --- | --- | --- | --- | --- | --- | --- | --- | --- | --- |
| **1** |  | 0.999811 | 0.999634 | 0.9484791 | 1 | 0.3262886 | 0.4813325 | 0.9778220 | 0.2717797 | 0.1914107 | 0.3372830 | 0.9999989 |
| **2** | n.s. |  | 0.930930 | 0.6062837 | 0.9999941 | 0.0894641 | 0.1538688 | 0.7078640 | 0.0705504 | 0.0456475 | 0.0934919 | 1 |
| **3** | n.s. | n.s. |  | 0.9999275 | 0.9968091 | 0.7801054 | 0.9052840 | 0.9999962 | 0.7142924 | 0.5877816 | 0.7917708 | 0.9840149 |
| **4** | n.s. | n.s. | n.s. |  | 0.8819913 | 0.9844004 | 0.9981895 | 1 | 0.9696365 | 0.9222178 | 0.9864181 | 0.7779825 |
| **5** | n.s. | n.s. | n.s. | n.s. |  | 0.2316176 | 0.3605842 | 0.9367418 | 0.1891940 | 0.1292313 | 0.2403501 | 1 |
| **6** | n.s. | n.s. | n.s. | n.s. | n.s. |  | 1 | 0.9609756 | 1 | 1 | 1 | 0.1565157 |
| **7** | n.s. | n.s. | n.s. | n.s. | n.s. | n.s. |  | 0.9927867 | 0.9999996 | 0.9999680 | 1 | 0.2556655 |
| **8** | n.s. | n.s. | n.s. | n.s. | n.s. | n.s. | n.s. |  | 0.9339598 | 0.8610411 | 0.9650143 | 0.8591879 |
| **9** | n.s. | n.s. | n.s. | n.s. | n.s. | n.s. | n.s. | n.s. |  | 1 | 1 | 0.1256769 |
| **10** | n.s. | * | n.s. | n.s. | n.s. | n.s. | n.s. | n.s. | n.s. |  | 0.9999999 | 0.0835966 |
| **11** | n.s. | n.s. | n.s. | n.s. | n.s. | n.s. | n.s. | n.s. | n.s. | n.s. |  | 0.1629717 |
| **14** | n.s. | n.s. | n.s. | n.s. | n.s. | n.s. | n.s. | n.s. | n.s. | n.s. | n.s. |  |

Tab. 14: p-adj.-values of ANOVA to compare expression levels of *col1a* between developmental stages. N.s. signifies p > 0.05 ; * = p ≤ 0.05 ; ** = p ≤ 0.01 ; *** = p ≤ 0.001.

| **Stage** | **1** | **2** | **3** | **4** | **5** | **6** | **7** | **8** | **9** | **10** | **11** | **14** |
| --- | --- | --- | --- | --- | --- | --- | --- | --- | --- | --- | --- | --- |
| **1** |  | 0.999545 | 0.9669613 | 0.9993699 | 0.6115278 | 0.9981866 | 0.9995586 | 0.1155551 | 0.8389935 | 1 | 1 | 0.3228539 |
| **2** | n.s. | n.s. | 0.9999886 | 1 | 0.9646866 | 1 | 1 | 0.4341659 | 0.3774150 | 0.9997315 | 0.9971442 | 0.0763996 |
| **3** | n.s. | n.s. |  | 0.9999934 | 0.9994710 | 0.9999993 | 0.9999880 | 0.7631045 | 0.1531655 | 0.9737602 | 0.9247594 | 0.0236458 |
| **4** | n.s. | n.s. | n.s. |  | 0.9695194 | 1 | 1 | 0.4508272 | 0.3621569 | 0.9996186 | 0.9963537 | 0.0720377 |
| **5** | n.s. | n.s. | n.s. | n.s. |  | 0.9829897 | 0.9642293 | 0.9931379 | 0.0290663 | 0.6385570 | 0.4988715 | 0.0036359 |
| **6** | n.s. | n.s. | n.s. | n.s. | n.s. |  | 1 | 0.5127816 | 0.3101748 | 0.9988140 | 0.9918984 | 0.0581077 |
| **7** | n.s. | n.s. | n.s. | n.s. | n.s. | n.s. |  | 0.4326899 | 0.3787953 | 0.9997400 | 0.9972073 | 0.0768007 |
| **8** | n.s. | n.s. | n.s. | n.s. | n.s. | n.s. | n.s. |  | 0.0023415 | 0.1256799 | 0.0804614 | 0.0002731 |
| **9** | n.s. | n.s. | n.s. | n.s. | * | n.s. | n.s. | ** |  | 0.8181147 | 0.9116257 | 0.9985891 |
| **10** | n.s. | n.s. | n.s. | n.s. | n.s. | n.s. | n.s. | n.s. | n.s. |  | 1 | 0.3018816 |
| **11** | n.s. | n.s. | n.s. | n.s. | n.s. | n.s. | n.s. | n.s. | n.s. | n.s. |  | 0.4196244 |
| **14** | n.s. | n.s. | ** | n.s. | ** | n.s. | n.s. | *** | n.s. | n.s. | n.s. |  |

Tab. 15: p-adj.-values of ANOVA to compare expression levels of *mustn1* between developmental stages. N.s. signifies p > 0.05 ; * = p ≤ 0.05 ; ** = p ≤ 0.01 ; *** = p ≤ 0.001.

| **Stage** | **1** | **2** | **3** | **4** | **5** | **6** | **7** | **8** | **9** | **10** | **11** | **14** |
| --- | --- | --- | --- | --- | --- | --- | --- | --- | --- | --- | --- | --- |
| **1** |  | 0.999020 | 1 | 0.9832827 | 0.4812594 | 0.2228676 | 0.1145096 | 0.4415421 | 0.0022976 | 0.1791927 | 0.7203279 | 0.6658563 |
| **2** | n.s. |  | 0.9906462 | 0.9999999 | 0.9326659 | 0.6957999 | 0.4723587 | 0.9123040 | 0.0173735 | 0.6196594 | 0.9921455 | 0.9854983 |
| **3** | n.s. | n.s. |  | 0.9358425 | 0.3341589 | 0.1383961 | 0.0672607 | 0.3014634 | 0.0012224 | 0.1089622 | 0.5581544 | 0.5021426 |
| **4** | n.s. | n.s. |  |  | 0.9898666 | 0.8774641 | 0.6899494 | 0.9843138 | 0.0381926 | 0.8219809 | 0.9997335 | 0.9992312 |
| **5** | n.s. | n.s. | n.s. | n.s. |  | 0.9999934 | 0.9988582 | 1 | 0.3317467 | 0.9999425 | 0.9999996 | 1 |
| **6** | n.s. | n.s. | n.s. | n.s. | n.s. |  | 0.9999998 | 0.9999984 | 0.6337129 | 1 | 0.9985130 | 0.9994265 |
| **7** | n.s. | n.s. | n.s. | n.s. | n.s. | n.s. |  | 0.9994444 | 0.8374683 | 1 | 0.9774065 | 0.9869752 |
| **8** | n.s. | n.s. | n.s. | n.s. | n.s. | n.s. | n.s. |  | 0.3663596 | 0.9999809 | 0.9999981 | 0.9999998 |
| **9** | ** | * | ** | * | n.s. | n.s. | n.s. | n.s. |  | 0.7092726 | 0.1732188 | 0.2036343 |
| **10** | n.s. | n.s. | n.s. | n.s. | n.s. | n.s. | n.s. | n.s. | n.s. |  | 0.9955291 | 0.9979585 |
| **11** | n.s. | n.s. | n.s. | n.s. | n.s. | n.s. | n.s. | n.s. | n.s. | n.s. |  | 1 |
| **14** | n.s. | n.s. | n.s. | n.s. | n.s. | n.s. | n.s. | n.s. | n.s. | n.s. | n.s. |  |

Tab. 16: p-adj.-values of ANOVA to compare expression levels of *sparc* between developmental stages. N.s. signifies p > 0.05 ; * = p ≤ 0.05 ; ** = p ≤ 0.01 ; *** = p ≤ 0.001.

| **Stage** | **1** | **2** | **3** | **4** | **5** | **6** | **7** | **8** | **9** | **10** | **11** | **14** |
| --- | --- | --- | --- | --- | --- | --- | --- | --- | --- | --- | --- | --- |
| **1** |  | 1 | 0.9999897 | 0.8260180 | 1 | 0.9998716 | 0.9879694 | 0.9999463 | 1 | 1 | 1 | 0.0001406 |
| **2** | n.s. |  | 0.9991707 | 0.6521580 | 0.9999997 | 0.9963725 | 0.9377017 | 0.9978442 | 1 | 0.9999988 | 1 | 0.0002926 |
| **3** | n.s. | n.s. |  | 0.9816667 | 0.9999976 | 1 | 0.9999533 | 1 | 0.9999111 | 0.9999994 | 0.9996760 | 0.0000398 |
| **4** | n.s. | n.s. | n.s. |  | 0.8587109 | 0.9935594 | 0.9999664 | 0.9901296 | 0.7580305 | 0.8819055 | 0.7020194 | 0.0000025 |
| **5** | n.s. | n.s. | n.s. | n.s. |  | 0.9999553 | 0.9926555 | 0.9999836 | 1 | 1 | 1 | 0.0001189 |
| **6** | n.s. | n.s. | n.s. | n.s. | n.s. |  | 0.9999974 | 1 | 0.9993549 | 0.9999825 | 0.9982560 | 0.0000278 |
| **7** | n.s. | n.s. | n.s. | n.s. | n.s. | n.s. |  | 0.9999913 | 0.9737294 | 0.9952057 | 0.9569330 | 0.0000095 |
| **8** | n.s. | n.s. | n.s. | n.s. | n.s. | n.s. | n.s. |  | 0.9996774 | 0.9999943 | 0.9990348 | 0.0000319 |
| **9** | n.s. | n.s. | n.s. | n.s. | n.s. | n.s. | n.s. | n.s. |  | 1 | 1 | 0.0001912 |
| **10** | n.s. | n.s. | n.s. | n.s. | n.s. | n.s. | n.s. | n.s. | n.s. |  | 0.9999999 | 0.0001042 |
| **11** | n.s. | n.s. | n.s. | n.s. | n.s. | n.s. | n.s. | n.s. | n.s. | n.s. |  | 0.0002406 |
| **14** | *** | *** | *** | *** | *** | *** | *** | *** | *** | *** | *** |  |

Tab. 17: p-adj.-values of ANOVA to compare expression levels of *igf1 x1* between developmental stages. N.s. signifies p > 0.05 ; * = p ≤ 0.05 ; ** = p ≤ 0.01 ; *** = p ≤ 0.001.

| **Stage** | **1** | **2** | **3** | **4** | **5** | **6** | **7** | **8** | **9** | **10** | **11** | **14** |
| --- | --- | --- | --- | --- | --- | --- | --- | --- | --- | --- | --- | --- |
| **1** |  | 0.999638 | 0.9840619 | 0.9975361 | 0.2474091 | 0.9905825 | 0.9999986 | 0.8593875 | 0.9036712 | 0.9999995 | 1 | 0.4725015 |
| **2** | n.s. |  | 0.9999989 | 1 | 0.6795003 | 0.9999999 | 0.9999999 | 0.9982245 | 0.4790861 | 0.9872722 | 0.9994682 | 0.1359020 |
| **3** | n.s. | n.s. |  | 1 | 0.8981167 | 1 | 0.9996721 | 0.9999962 | 0.2539123 | 0.8955181 | 0.9805664 | 0.0567663 |
| **4** | n.s. | n.s. | n.s. |  | 0.7853195 | 1 | 0.9999933 | 0.9997676 | 0.3738050 | 0.9635065 | 0.9967171 | 0.0949119 |
| **5** | n.s. | n.s. | n.s. | n.s. |  | 0.8666900 | 0.4745513 | 0.9916480 | 0.0091757 | 0.1204195 | 0.2341476 | 0.0014277 |
| **6** | n.s. | n.s. | n.s. | n.s. | n.s. |  | 0.9998857 | 0.9999819 | 0.2891683 | 0.9224582 | 0.9882328 | 0.0671579 |
| **7** | n.s. | n.s. | n.s. | n.s. | n.s. | n.s. |  | 0.9789360 | 0.6840818 | 0.9992262 | 0.9999971 | 0.2460008 |
| **8** | n.s. | n.s. | n.s. | n.s. | n.s. | n.s. | n.s. |  | 0.1041039 | 0.6415544 | 0.8447495 | 0.0194103 |
| **9** | n.s. | n.s. | n.s. | n.s. | ** | n.s. | n.s. | n.s. |  | 0.9861883 | 0.9147186 | 0.9995875 |
| **10** | n.s. | n.s. | n.s. | n.s. | n.s. | n.s. | n.s. | n.s. | n.s. |  | 0.9999998 | 0.7172713 |
| **11** | n.s. | n.s. | n.s. | n.s. | n.s. | n.s. | n.s. | n.s. | n.s. | n.s. |  | 0.4922707 |
| **14** | n.s. | n.s. | n.s. | n.s. | ** | n.s. | n.s. | * | n.s. | n.s. | n.s. |  |

Tab. 18: p-adj.-values of ANOVA to compare expression levels of *igf1ra* between developmental stages. N.s. signifies p > 0.05 ; * = p ≤ 0.05 ; ** = p ≤ 0.01 ; *** = p ≤ 0.001.

| **Stage** | **1** | **2** | **3** | **4** | **5** | **6** | **7** | **8** | **9** | **10** | **11** | **14** |
| --- | --- | --- | --- | --- | --- | --- | --- | --- | --- | --- | --- | --- |
| **1** |  | 1 | 0.9926876 | 0.9242921 | 0.9995190 | 0.0180718 | 0.0289865 | 0.3893607 | 0.1954091 | 0.1396250 | 0.0161076 |  |
| **2** | n.s. |  | 0.9979855 | 0.9618512 | 0.9999421 | 0.0253586 | 0.0403438 | 0.4774046 | 0.2535183 | 0.1846101 | 0.0226399 |  |
| **3** | n.s. | n.s. |  | 0.9999964 | 0.9999999 | 0.1469148 | 0.2154359 | 0.9299681 | 0.7365321 | 0.6244361 | 0.1334278 |  |
| **4** | n.s. | n.s. | n.s. |  | 0.9995852 | 0.3030663 | 0.4144463 | 0.9936767 | 0.9267175 | 0.8584882 | 0.2794324 |  |
| **5** | n.s. | n.s. | n.s. | n.s. |  | 0.0842843 | 0.1280287 | 0.8156519 | 0.5605745 | 0.4479719 | 0.0759757 |  |
| **6** | * | * | n.s. | n.s. | n.s. |  | 1 | 0.8620198 | 0.9791633 | 0.9940607 | 1 |  |
| **7** | * | * | n.s. | n.s. | n.s. | n.s. |  | 0.9371686 | 0.9952800 | 0.9991846 | 1 |  |
| **8** | n.s. | n.s. | n.s. | n.s. | n.s. | n.s. | n.s. |  | 0.9999954 | 0.9998765 | 0.8387970 |  |
| **9** | n.s. | n.s. | n.s. | n.s. | n.s. | n.s. | n.s. | n.s. |  | 1 | 0.9721101 |  |
| **10** | n.s. | n.s. | n.s. | n.s. | n.s. | n.s. | n.s. | n.s. | n.s. |  | 0.9912724 |  |
| **11** | * | * | n.s. | n.s. | n.s. | n.s. | n.s. | n.s. | n.s. | n.s. |  |  |
| **14** |  |  |  |  |  |  |  |  |  |  |  |  |

Tab. 19: p-adj.-values of ANOVA to compare expression levels of *gh* between developmental stages. N.s. signifies p > 0.05 ; * = p ≤ 0.05 ; ** = p ≤ 0.01 ; *** = p ≤ 0.001.

| **Stage** | **1** | **2** | **3** | **4** | **5** | **6** | **7** | **8** | **9** | **10** | **11** | **14** |
| --- | --- | --- | --- | --- | --- | --- | --- | --- | --- | --- | --- | --- |
| **1** |  | 0.0943652 | 0.0089328 | 0.0206449 | 0.0053822 | 0.0151919 | 0.6452482 | 0.9934075 | 0.9039056 | 0.0028030 | 0.0046350 | 0.1337845 |
| **2** | n.s. |  | 0.9936743 | 0.9998438 | 0.9744596 | 0.9992048 | 0.9805903 | 0.0088189 | 0.0026773 | 0.0000013 | 0.0000020 | 0.0000642 |
| **3** | ** | n.s. |  | 0.9999998 | 1 | 1 | 0.5059720 | 0.0006882 | 0.0002064 | 0.0000001 | 0.0000002 | 0.0000055 |
| **4** | * | n.s. | n.s. |  | 0.9999779 | 1 | 0.7296984 | 0.0016467 | 0.0004920 | 0.0000003 | 0.0000004 | 0.0000125 |
| **5** | ** | n.s. | n.s. | n.s. |  | 0.9999985 | 0.3806035 | 0.0004107 | 0.0001238 | 0.0000001 | 0.0000001 | 0.0000034 |
| **6** | * | n.s. | n.s. | n.s. | n.s. |  | 0.6486474 | 0.0011927 | 0.0003566 | 0.0000002 | 0.0000003 | 0.0000093 |
| **7** | n.s. | n.s. | n.s. | n.s. | n.s. | n.s. |  | 0.1296402 | 0.0463073 | 0.0000201 | 0.0000327 | 0.0012246 |
| **8** | n.s. | ** | *** | ** | *** | ** | n.s. |  | 0.9999946 | 0.0339315 | 0.0536348 | 0.6554309 |
| **9** | n.s. | ** | *** | *** | *** | *** | * | n.s. |  | 0.0980612 | 0.1476429 | 0.9090932 |
| **10** | ** | *** | *** | *** | *** | *** | *** | n.s. | n.s. |  | 1 | 0.8386780 |
| **11** | ** | *** | *** | *** | *** | *** | *** | n.s. | n.s. | n.s. |  | 0.9210770 |
| **14** | n.s. | *** | *** | *** | *** | *** | *** | n.s. | n.s. | n.s. | n.s. |  |


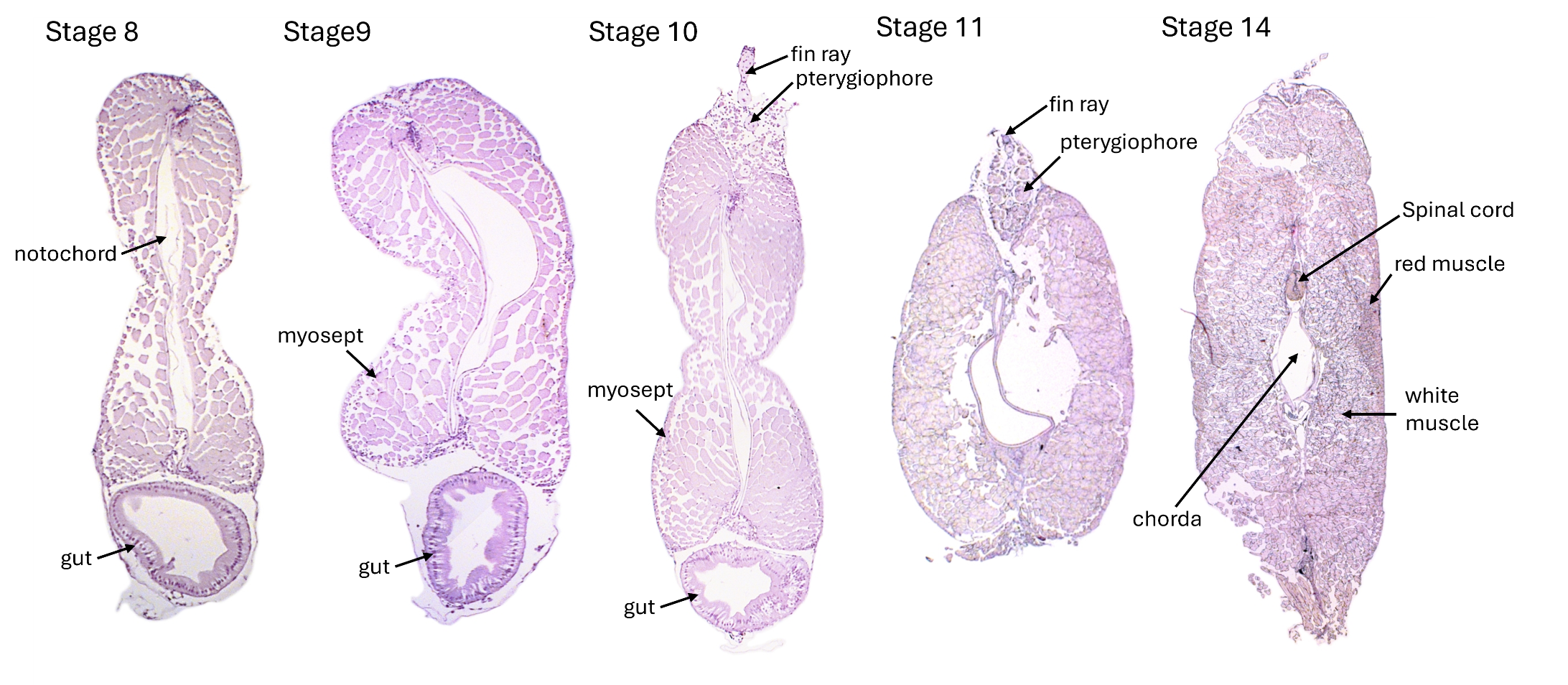
Fig. 1: HE stained Histological cross sections of herring larvae in stages 8-11 and 14. The arrows mark important structures such as the gut, notochord, red and white muscle, myosepta, fin rays & pterygiophores and spinal cord and chorda.


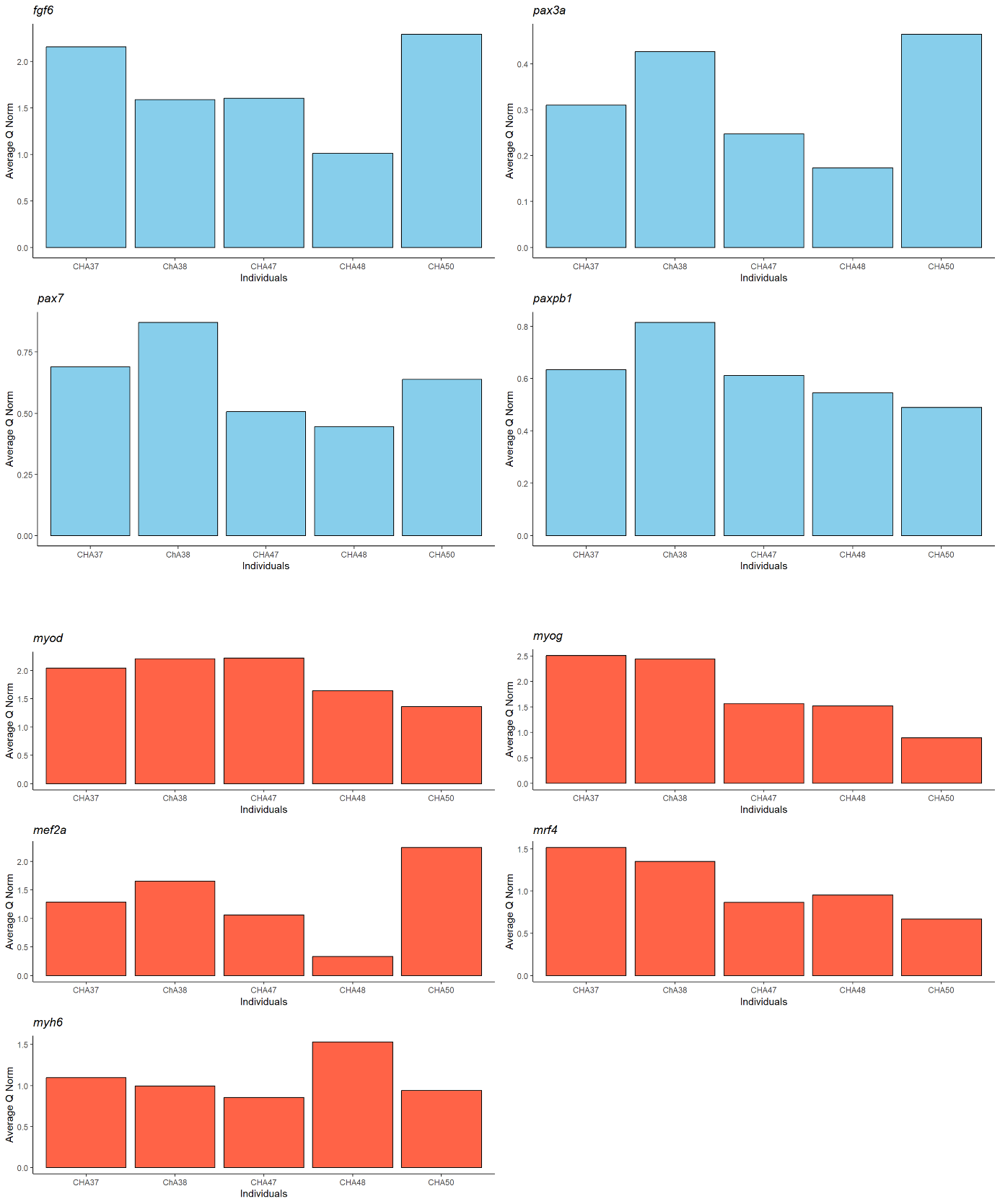


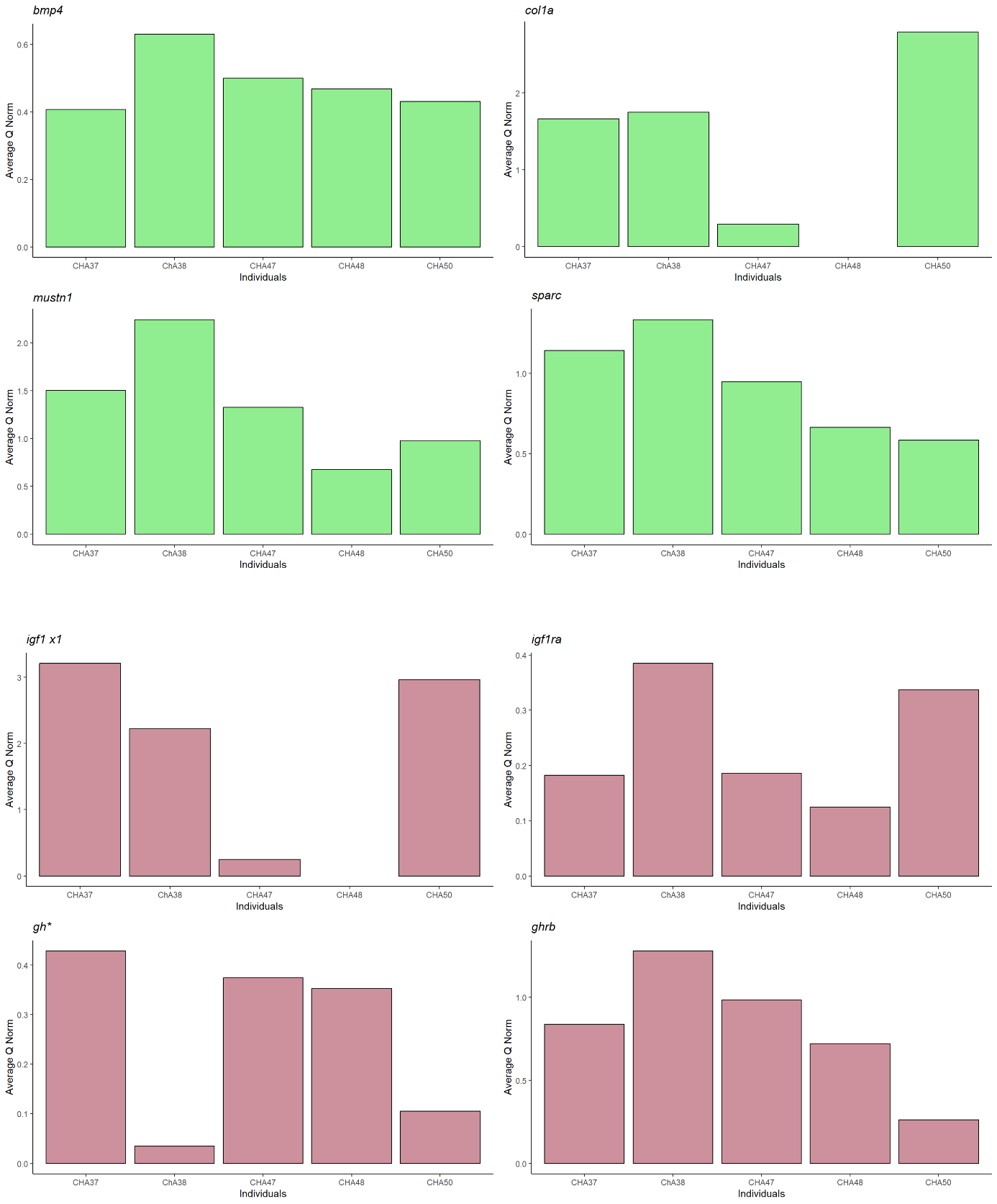


Fig. 2: : Expression of genes related to general developmental processes (*fgf6, pay3a, pax7 & paxbp1*), muscle development (*myod, myog, mef2a, mrf4, myh6*), skeletal or structural development (*bmp4, col1a, mustn1, sparc*) and growth (*igf1 x1, igf1ra, gh, ghrb)* in individual larvae of the pelvic fin developmental phase. The bars indicate the normed quantity of detected cDNA per individual.
